# Pulmonary osteoclast-like cells in silica induced pulmonary fibrosis

**DOI:** 10.1126/sciadv.adl4913

**Published:** 2024-07-10

**Authors:** Yoshihiro Hasegawa, Jennifer M. Franks, Yusuke Tanaka, Yasuaki Uehara, David F. Read, Claire Williams, Sanjay Srivatsan, Lori B. Pitstick, Nikolaos M. Nikolaidis, Ciara M. Shaver, Jonathan Kropski, Lorraine B. Ware, Chase J. Taylor, Nicholas E. Banovich, Huixing Wu, Jason C. Gardner, Andrew R. Osterburg, Jane J. Yu, Elizabeth J. Kopras, Steven L. Teitelbaum, Kathryn A. Wikenheiser-Brokamp, Cole Trapnell, Francis X. McCormack

**Affiliations:** ^1^ Division of Pulmonary, Critical Care and Sleep Medicine, Department of Internal Medicine, University of Cincinnati, Cincinnati, OH, USA.; ^2^ Department of Genome Sciences, University of Washington, Seattle, WA, USA.; ^3^ Division of Allergy, Pulmonary, and Critical Care Medicine, Department of Medicine, Vanderbilt University Medical Center, Nashville, TN, USA.; ^4^ Department of Pathology and Immunology, and Division of Bone and Mineral Diseases, Department of Medicine, Washington University School of Medicine, St. Louis, MO, USA.; ^5^ Division of Pathology and Laboratory Medicine and Perinatal Institute, Division of Pulmonary Biology, Cincinnati Children’s Hospital Medical Center, Cincinnati, OH, USA.; ^6^ Department of Pathology and Laboratory Medicine, University of Cincinnati, Cincinnati, OH, USA.

## Abstract

The pathophysiology of silicosis is poorly understood, limiting development of therapies for those who have been exposed to the respirable particle. We explored mechanisms of silica-induced pulmonary fibrosis in human lung samples collected from patients with occupational exposure to silica and in a longitudinal mouse model of silicosis using multiple modalities including whole-lung single-cell RNA sequencing and histological, biochemical, and physiologic assessments. In addition to pulmonary inflammation and fibrosis, intratracheal silica challenge induced osteoclast-like differentiation of alveolar macrophages and recruited monocytes, driven by induction of the osteoclastogenic cytokine, receptor activator of nuclear factor κΒ ligand (RANKL) in pulmonary lymphocytes, and alveolar type II cells. Anti-RANKL monoclonal antibody treatment suppressed silica-induced osteoclast-like differentiation in the lung and attenuated pulmonary fibrosis. We conclude that silica induces differentiation of pulmonary osteoclast-like cells leading to progressive lung injury, likely due to sustained elaboration of bone-resorbing proteases and hydrochloric acid. Interrupting osteoclast-like differentiation may therefore constitute a promising avenue for moderating lung damage in silicosis.

## Teaser

Silica induces osteoclastic differentiation of recruited and resident myeloid cells which contribute to pulmonary fibrosis.

## INTRODUCTION

Crystalline silica is the most ubiquitous mineral on Earth (*1*–*4*). The inhalation of silica particles causes silicosis, a chronic interstitial lung disease that results in progressive pulmonary dysfunction (*1*–*4*). As one of the most important occupational diseases in both developing and developed nations, silicosis is estimated to impact 2.2 million workers in the United States (*5*), 2 million workers in European Union (*6*), and more than 23 million workers in China (*4*, *7*, *8*). Pathological subtypes of fibrosis in silicosis include simple nodular silicosis, progressive massive fibrosis, and diffuse interstitial fibrosis (*2*, *4*). Silica inhalation is also associated with several life-threatening comorbidities, such as acute silicoproteinosis, pulmonary tuberculosis, chronic obstructive pulmonary disease, and lung cancer (*1*, *4*, *9*, *10*). The pathogenesis of silicosis is complex but clearly involves engagement of macrophage scavenger receptors and activation of the inflammasome, release of reactive oxygen species, matrix remodeling enzymes, and proinflammatory and profibrotic cytokines and chemokines (*4*, *11*, *12*). The relative contribution of each of these pathogenic mechanisms to pulmonary fibrosis is not clear.

The advent of single-nucleus and single-cell RNA sequencing (snRNA-seq, scRNA-seq) provides an opportunity to dissect complex disease mechanisms at levels of resolution that have not previously been possible. Here, we use high-throughput genomic sequencing in combination with multiple experimental methodologies to perform an extensive study of silica-induced pathology in fibrotic human lungs and longitudinally collected whole mouse lungs. We report evidence of silica-induced osteoclastic differentiation of pulmonary monocytes and macrophages that contributes to progressive lung fibrosis.

Osteoclasts are multinucleated giant cells (MNGCs) that mediate bone resorption and maintain normal bone health. MNGC originating from hematopoietic, myeloid, multipotential precursors differentiate into mature osteoclasts under the influence of two cytokines: macrophage colony-stimulating factor (M-CSF; *CSF1*) and receptor activator of nuclear factor κB ligand (RANKL; *TNFSF11*) (*13*–*16*). Inflammatory cytokines, including tumor necrosis factor–α (TNF-α), interleukin-1β (IL-1β), and IL-6 (*17*, *18*), can also contribute to osteoclast formation and function. Typically, osteoclasts attach to bone, assemble cytoskeletal actin rings, and seal off an area through binding of integrin β3 (ITGB3) to osteopontin (SPP1) and other bone ligands. Active secretion of hydrochloric acid into the lacunar space beneath the cell by adenosine triphosphatase H^+^ transporting v0 subunit d2 (ATP6V0D2) and chloride voltage-gated channel 7 degrades the mineral components of bone and exposes the bone matrix to cosecreted collagen and elastin degrading proteases such as matrix metalloproteinase 9 (MMP-9) and cathepsin K (CTSK) (*19*–*22*). Osteoclasts also express tartrate-resistant acid phosphatase (TRAP), which plays critical roles in facilitating osteoclast migration across the bone surface (*23*) and creating reactive oxygen species that resorb and degrade bone (*24*).

Previously, we identified osteoclast-like differentiation of lung mononuclear cells in pulmonary alveolar microlithiasis, in which alveolar macrophages (AMs) adopt an osteoclastic phenotype promoting clearance of alveolar hydroxyapatite Ca_5_(PO_4_)_3_(OH) microliths (*25*, *26*). From this work, we postulated that other particulates may induce osteoclast formation in the lung and that sustained osteoclastic activity may prove pathogenic. Osteoclasts are not normally found in the lungs, and no homeostatic role has been ascribed to this cell type in the pulmonary context. Osteoclast-like cells have recently been reported in silicotic rats (*27*), but no comprehensive study has yet demonstrated the specific existence of pulmonary osteoclasts in human samples, analyzed molecular pathways of pulmonary osteoclastic activation, nor evaluated osteoclastic contribution to pulmonary fibrosis. Here, we characterize silica-induced osteoclastic transformation of pulmonary macrophages and propose a novel mechanism for pulmonary fibrosis driven by RANKL-dependent osteoclast differentiation.

## RESULTS

### Pulmonary inflammation and fibrosis in human and murine silicosis

**Fig. 1.**
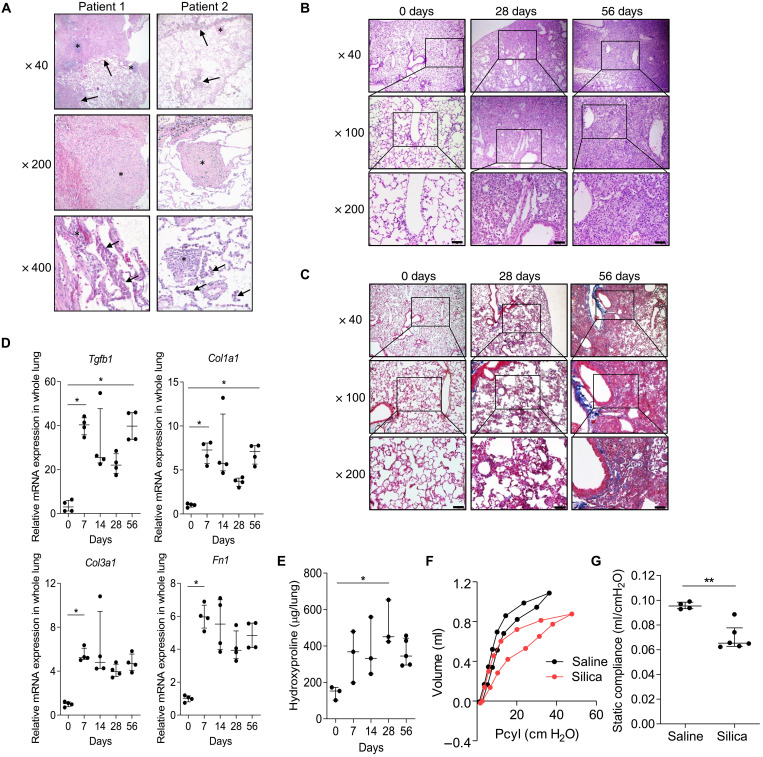
Silicosis features of progressive fibrosis, silicotic nodules, and infiltrating monocytes are recapitulated in the mouse model. (**A**) H&E-stained human silicotic lung sections. Patient 1 pathologic features include extensive fibrosis (arrows, top) with lymphocyte predominant inflammation (*, top), collagenized silicotic nodules (*, middle), surrounding alveoli with septal inflammation (*, bottom), and intra-AM accumulations (arrows, bottom). Pathologic features in patient 2 include focal fibrosis with prominent dust macules and accumulations of pigment containing macrophages (arrows, top), rare small collagenized silicotic nodules (*, top and middle), and alveoli with lymphocyte predominant septal inflammation (*, bottom), and intra-AM aggregates (arrows, bottom). (**B**) H&E and (**C**) Masson’s trichrome reagent–stained lung sections collected from C57BL/6J silica-challenged mice (5 mg, i.t.) at d0 (pre-exposure), d28, and d56. Scale bars, 50 μm. (**D**) RT-qPCR of *Tgfb1*, *Col1a1*, *Col3a1*, and *Fn1* from whole lung homogenates at indicated days after silica administration (5 mg i.t.) (*N* = 4 mice per group). (**E**) Hydroxyproline levels in whole lungs were quantified (*N* = 3 to 5 mice per group). (**F**) Pulmonary function tests at d56 postsilica challenge (5 mg, i.t.). The representative curves of pressure in the cylinder (Pcyl)/volume from the two groups and (**G**) compliance measurements were made using the forced oscillation method (see Materials and Methods) (*N* = 4 to 6 mice per group). Error bars show median with interquartile range. *P < 0.05 and **P < 0.01 by Mann-Whitney *U* test in two groups comparison and Kruskal-Wallis test followed by Dunn’s test in multiple comparisons.

Inhalation of silica particles is known to result in pulmonary inflammation and fibrosis to varying pathogenic degrees. Histopathology of late-stage lung disease often features extensive fibrosis, collagenized silicotic nodules, and patchy chronic inflammation (shown in patient 1, Fig. 1A), while other cases may exhibit less extensive fibrosis with prominent dust macules representing a common reaction to inhaled dust along with rare, small collagenized nodules (patient 2, Fig. 1A).

As a model of silicosis, we challenged male C57BL/6J mice with intratracheal (i.t.) silica (*28*–*33*). Hematoxylin and eosin (H&E) (Fig. 1B) and Masson trichrome staining (Fig. 1C) at 28 days (d28) and d56 postchallenge confirmed that i.t. silica induces patchy but extensive interstitial and intra-alveolar inflammation composed predominantly of lymphocytes and macrophages. The alveolar spaces were filled with abundant enlarged macrophages admixed with granular proteinaceous material. The inflammatory infiltrate was initially peribronchiolar and perivascular in distribution with progression to confluent lesions associated with collagen deposition by d56 posttreatment. Analysis of whole lung RNA isolated from silica-treated mice revealed a sustained increase in expression of multiple fibrosis-related genes over the 56-day time course including transforming growth factor–β1 (*Tgfb1*), collagen 1α-1 (*Col1a1*), collagen 3α-1 (*Col3a1*), and fibronectin-1 (*Fn1*) (Fig. 1D), as well as a durable increase in the hydroxyproline content of lung tissues (Fig. 1E). The pressure-volume (*P*-*V*) loop of the lungs was shifted down and to the right in silica-treated mice relative to mice treated with vehicle (Fig. 1F), consistent with a ~30% reduction in median static compliance (0.070 ml/cmH_2_O versus 0.096 ml/cmH_2_O, respectively) (Fig. 1G). These data demonstrate that silica induces a fibrogenic program and restrictive physiologic defect that mimics silica-induced pulmonary pathologies in humans based on gene expression, histological, biochemical, and physiological assessments.

### Single-cell analysis of the murine silicosis lung

**Fig. 2.**
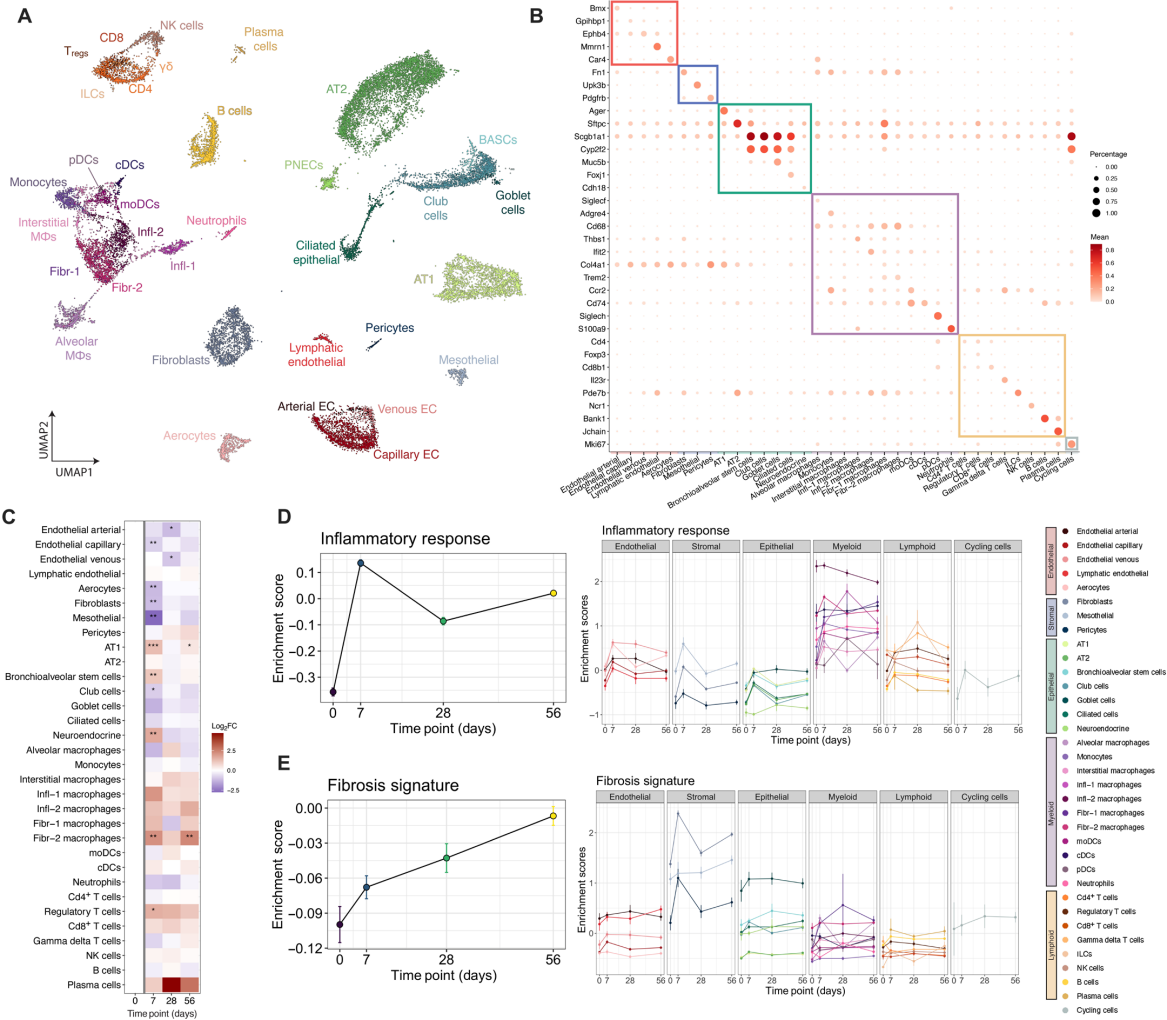
snRNA-seq depicts the global molecular dynamics in silicosis. snRNA-seq was performed on whole lung from i.t. silica-challenged mice at time 0, d7, d28, and d56. (**A**) Uniform Manifold Approximation Projection (UMAP) plot of all annotated cells, colored according to fine scale annotation. (**B**) Selected marker genes expression used for broad and fine-scale cell type annotation, colored by normalized expression, and size indicates percent cells of the cell type with detectable expression of the gene. (**C**) Cell state abundance relative to d0 shown over time. *P < 0.05, **P < 0.005, and ***P < 0.0005; (beta-binomial test, Benjamini-Hochberg correction). Gene set enrichment scores for inflammation (**D**) and fibrosis (**E**) shown over time summarized for all cells collected from each time point (left) and colored by individual cell state (right). T_regs_, regulatory T cell; FC, fold change; PNECs, pulmonary neuroendocrine cells; BASCs, bronchioalveolar stem cells; ILCs, innate immune cells; pDCs, plasmacytoid dendritic cells; cDCs, classical dendritic cells; EC, endothelial cells; moDC, monocyte-derived dendritic cells.

To further examine fibrogenic programs induced by silica, we performed a longitudinal transcriptomic analysis using snRNA-seq of the lungs from pre- and postsilica challenged mice. We recovered a total of 23,794 nuclei from 12 whole lung samples across four time points including d0 (pre-), d7, d28, and d56 days post-i.t. silica. Thirty-five unique cell states spanning epithelial, endothelial, stromal, myeloid, and lymphoid cell lineages were identified using highly and specifically expressed marker genes (Fig. 2, A and B, and figs. S1 and S2). Silicosis is known to produce both acute and chronic inflammation in the lung followed by progressive fibrosis. Gene set enrichment analysis revealed that inflammatory gene processes peak at d7 postexposure and chronically remain higher than baseline, while fibrotic gene expression processes continually increase following exposure (Fig. 2, C to E). Cell states uniquely varied both in the baseline activation of inflammatory and fibrotic gene signatures and in the subsequent temporal response following silica exposure. Many cell states were differentially abundant over time with most notable changes occurring at d7 postexposure (Fig. 2C). Alveolar type I cells, bronchioalveolar stem cells, regulatory T cells, and fibrotic (Fibr-2) macrophages significantly increased in relative abundance following silica exposure, while arterial, capillary, and venous endothelial cells, aerocytes, fibroblasts, mesothelial, and club cells decreased in relative abundance (adjusted *P* < 0.05, beta-binomial test). Neuroendocrine cells demonstrated a significant, yet transient, increase at d7 postsilica exposure, which may reflect a potential role in supporting acute inflammation (*34*).

**Fig. 3.**
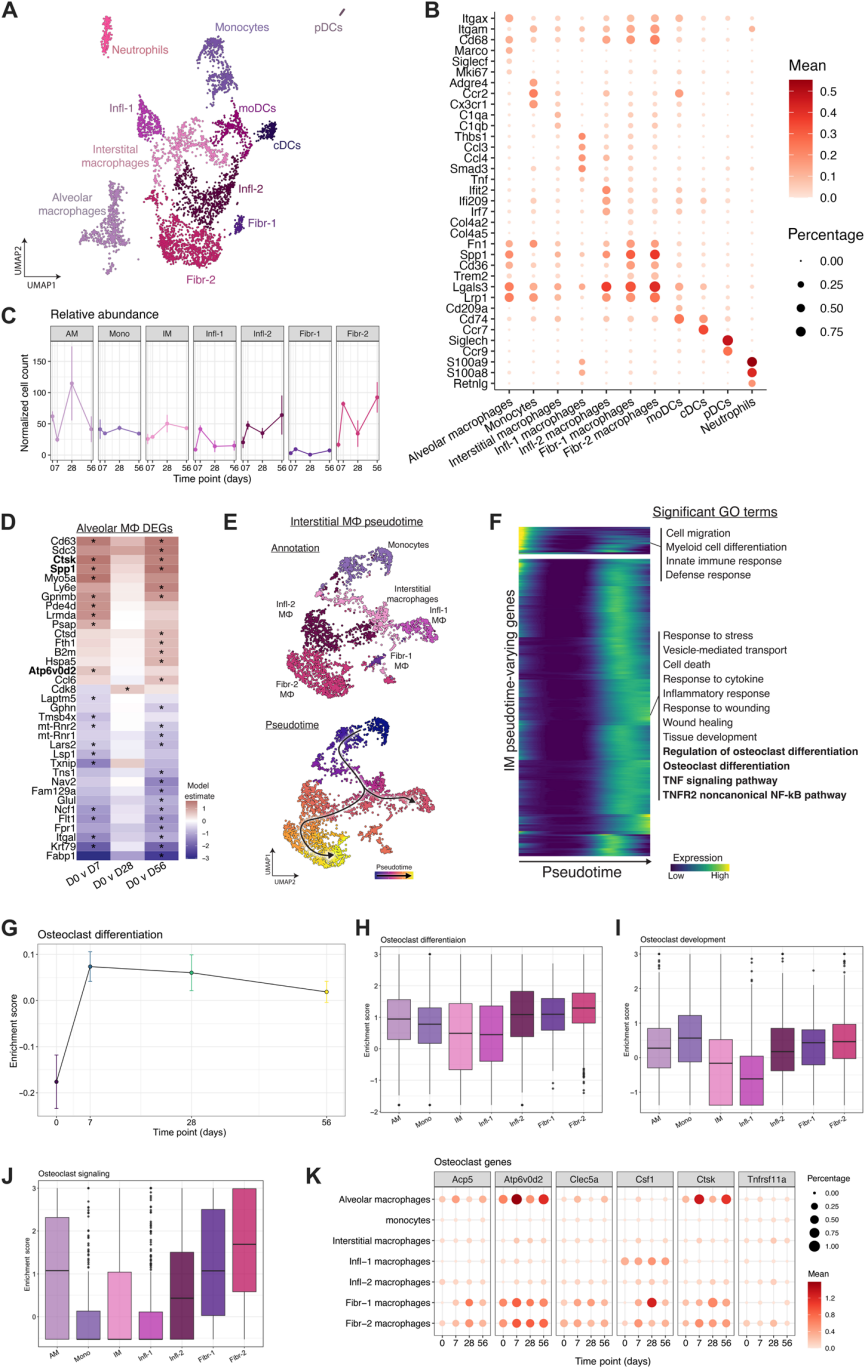
Myeloid cells demonstrate remarkable heterogeneity and activation of osteoclast-related transcriptional programs in silicosis. Analysis of myeloid populations identified in longitudinal snRNA-seq of whole lung from i.t. silica-challenged mice. (**A**) UMAP plot of myeloid cells. (**B**) Selected marker genes used for fine-scale cell annotation of myeloid cells. Size indicates percent of cells expressing the marker, and color indicates average expression value. (**C**) Relative abundance (normalized by library size factors) is shown for each myeloid cell state over time. (**D**) Differentially expressed genes (DEGs) relative to d0 were identified for AMs (false discovery rate). Osteoclast-related genes denoted in bold typeface. (**E**) UMAP of IMs colored according to fine-scale annotation (top) and pseudotime (bottom). (**F**) Heatmap of genes differentially expressed over pseudotime as IMs differentiate toward profibrotic phenotypes. Each row represents a gene, and color represents relative intensity of expression. Significant (g:Profiler, g:SCS) representative gene ontology (GO) terms shown on right. (**G**) Osteoclast differentiation enrichment scores for all myeloid cells plotted over time. Boxplots demonstrating cell state enrichment scores for (**H**) osteoclast differentiation, (**I**) osteoclast development, and (**J**) osteoclast signaling. (**K**) Select osteoclast genes plotted over time for myeloid cell states. *P < 0.05.

We further interrogated the myeloid cells to identify the genetic programs activated in this lineage following silica exposure in the mouse lung (Fig. 3A). Within the myeloid cells, we identified previously described populations of alveolar (SiglecF^+^) and interstitial (SiglecF^−^) macrophages as well as populations of dendritic cells and neutrophils (Fig. 3B). The relative abundance of myeloid subsets over time after silica challenge is shown in Fig. 3C. Interstitial macrophages (IMs) increased in the silicosis lung and demonstrated remarkable heterogeneity. Thus, IMs were subclustered and annotated into phenotypically descriptive groups (fig. S3). Monocytes represented the most recently recruited cells in the lung based on high expression of *Ccr2*. Transitional IMs express low levels of many proinflammatory and profibrotic genes and likely represent an intermediate cell state poised to adopt a more specialized macrophage phenotype. All macrophage subsets in our analysis showed some activation of both inflammatory and fibrotic gene processes, and the traditional M1/M2 nomenclature is insufficient to describe the heterogeneity evident in our dataset (fig. S3). Thus, we annotated two populations of proinflammatory macrophages (Infl-1 and Infl-2) and two populations of profibrotic macrophages (Fibr-1 and Fibr-2) based on gene expression and pathway activation differences. While both Infl-1 and Infl-2 demonstrated activation of TNF-related gene expression, Infl-2 also exhibited inflammatory gene expression signaling driven by interferon-γ (IFN-γ) (fig. S3). Infl-1 peaked at d7 and likely represents a population of myeloid cells responsible for acute inflammation in the silicosis lung, whereas Infl-2 macrophages contribute broad inflammatory signatures evident in chronic inflammation. Infl-2 macrophages also express many profibrotic genes and may serve as precursors to fibrotic macrophage cell states. Fibr-1 macrophages likely contribute to fibrosis due to high expression of tissue remodeling and fibrosis-related genes including *Col4a1*, *Fn1*, and *Osmr* (Fig. 3B). The Fibr-2 macrophage subset likely represents end-stage profibrotic macrophages in the silicosis lung due to waning expression of profibrotic genes coupled with increased expression of foamy macrophage markers including *Spp1*, *Cd36*, and lipid-metabolizing genes such as *Lgals3* and *Lrp1* (Fig. 3B). Many profibrotic genes including *Spp1*, *Lgals3*, and *Lrp1* are also highly expressed in AMs (Fig. 3B).

### Transcriptional activation of osteoclast gene programs in lung macrophages

To identify the silica-induced temporal changes in genetic programs of macrophages, we performed two analyses. First, in the *SiglecF^+^* AMs, we identified differentially expressed genes over time. Several osteoclast marker genes were significantly increased in the AMs of silicosis lungs compared to baseline including *Ctsk*, *Spp1*, and *Atp6v0d2* (Fig. 3D). To identify the genetic programs changing within the IMs (*SiglecF^−^*), we performed pseudotime analysis (Fig. 3E). Pseudotime analysis linked the progression of recently recruited interstitial monocytes to a transitional state followed by proinflammatory and profibrotic phenotypes. Genes with significantly varying expression along the pseudotime trajectory were identified and hierarchically clustered, and clusters were annotated on the basis of gene ontology (GO) (Fig. 3, E and F). Genes that showed increased expression later in pseudotime were significantly enriched for inflammatory response, wound healing, tissue development, osteoclast differentiation, and TNF signaling pathway (adjusted *P* < 0.05, g:Profiler). Given that osteoclastic gene expression was identified in both interstitial and AMs, we calculated enrichment scores using previously published lists of genes (*35*, *36*) associated with osteoclast differentiation, development, and signaling for each macrophage subtype (Fig. 3, G to J). The activation levels of these signatures increase markedly postsilica exposure, and the highest enrichment scores were present in AMs and Fibr-2 macrophages. Moreover, these populations expressed high levels of osteoclast markers including *Acp5*, *Atp6v062*, *Clec5a*, *Ctsk*, *Csf1r*, and modest levels of *Tnfrsf11a* (Fig. 3K).

In summary, both tissue-resident AMs and infiltrating bone marrow–derived macrophages demonstrate robust genomic signatures of osteoclastic transformation following silica exposure. This supports the hypothesis that microenvironmental signals in the silica-challenged lung are critical in shaping pulmonary macrophage polarization states and functional phenotype.

### Single-cell analysis of human coal worker lungs

**Fig. 4.**
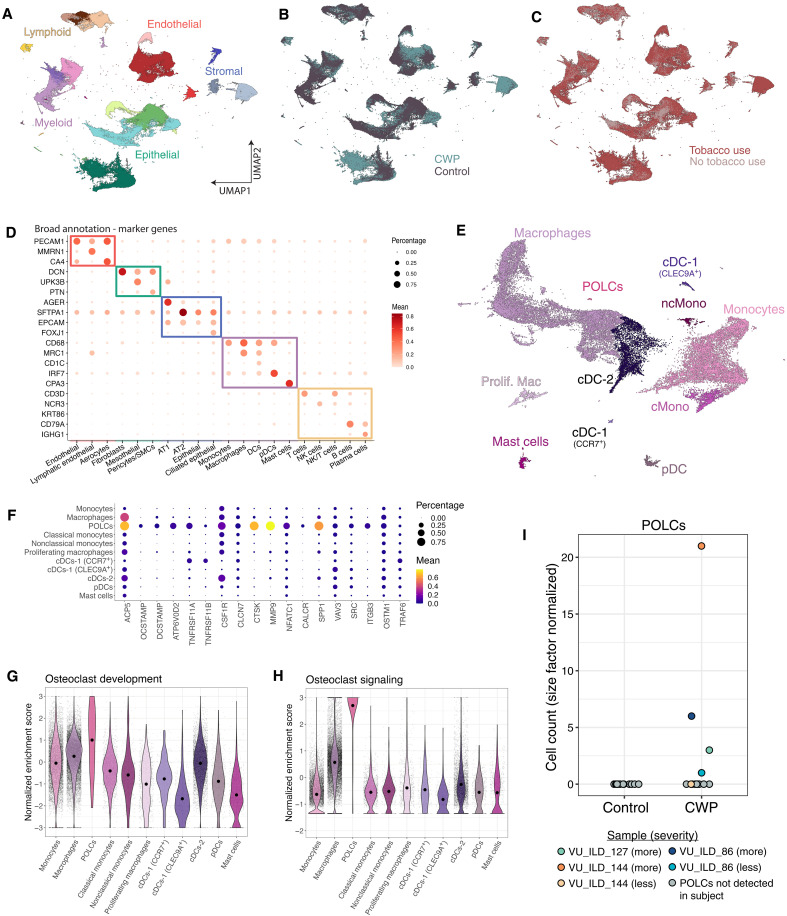
Single-cell analysis of coal miner lungs reveals a population of POLCs. UMAP of annotated cell types colored by (**A**) fine annotation, (**B**) case-control status, and (**C**) patient-reported tobacco use. (**D**) Cells were broadly and finely annotated using canonical marker genes. (**E**) UMAP of myeloid cells. (**F**) Osteoclast-related gene expression for all myeloid cells, colored by normalized average expression and sized according to percent cells expressing the marker. (**G**) Osteoclast development and (**H**) osteoclast signaling enrichment scores for each cell grouped by annotation. (**I**) Normalized cell counts of POLCs per subject. POLC, pulmonary osteoclast like cells; SMC, smooth muscle cells; CWP, coal workers pneumoconiosis.

In addition to carbon dust, coal miners are exposed to high concentrations of respirable silica and are at risk for silicosis. We postulated that explanted lungs from coal miners undergoing transplantation may also exhibit osteoclastic gene signatures. scRNA-seq was performed on lung tissue collected from lung explants from 9 coal workers with pulmonary fibrosis and 12 nonfibrotic age-, sex-, and smoking status–matched controls. We recovered 214,448 cells from 26 samples (tables S1 and S2 and fig. S4) and used highly and specifically expressed cell markers to annotate 37 unique cell states across immune, stromal, epithelial, and endothelial cell lineages (Fig. 4, A to D, and fig. S5). Within the myeloid cells, we identified a small but distinct population of pulmonary osteoclast-like cells (POLCs), expressing high levels of many osteoclast marker genes including *SPP1*, *ACP5*, *CTSK*, and moderate levels of *ATP6V0A1*, *OCSTAMP*, and *DCSTAMP* (Fig. 4, E and F). We quantified enrichment scores for osteoclast development and signaling in myeloid cells using published gene sets. POLCs demonstrate significantly higher enrichment scores for both osteoclast development and osteoclast signaling compared to all other myeloid cells (Fig. 4, G and H) [adjusted *P* < 0.05, analysis of variance (ANOVA) Tukey]. POLCs are present in four fibrotic samples (three of nine coal miner patients) and none of the control samples (Fig. 4I). Moreover, we sought to identify the myeloid populations that may be giving rise to POLCs by identifying differentially expressed genes and pathways between cases and controls. We identified osteoclast differentiation as a significantly enriched pathway among the differentially expressed genes identified multiple myeloid populations including monocytes, classical monocytes, and nonclassical monocytes (fig. S6). We conclude that POLCs show a definitive correlation with silica exposure and subsequent fibrosis, regardless of prior tobacco usage.

### Osteoclast-like cells are present in the lungs of patients with silicosis and in silicosis mouse models

**Fig. 5.**
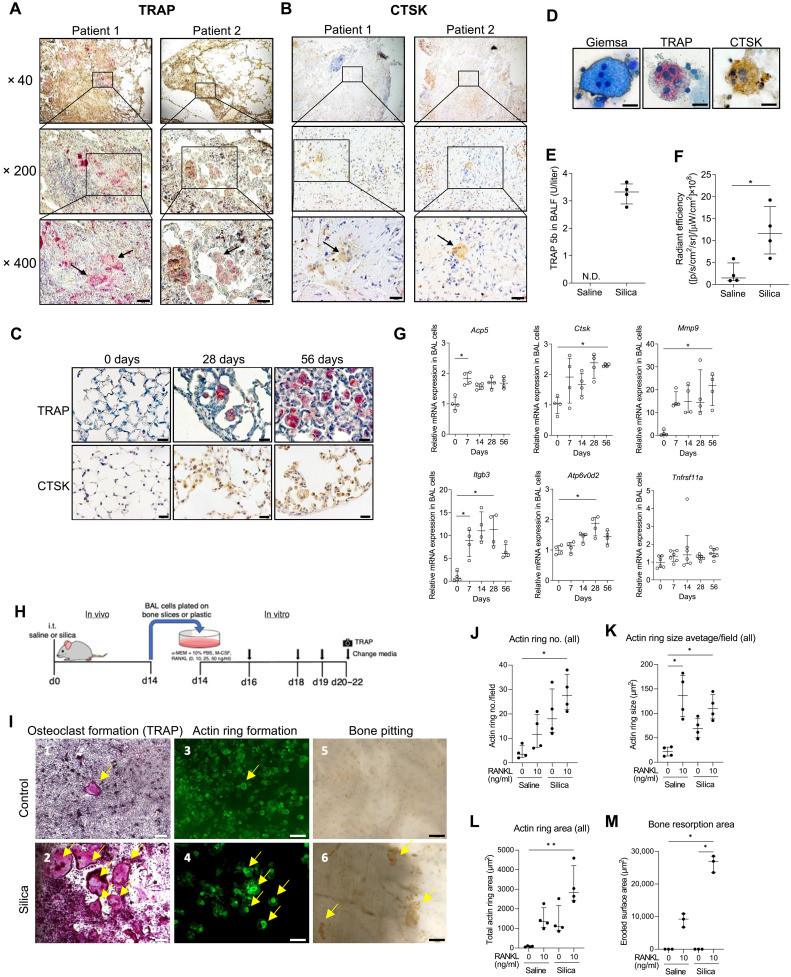
Prominent osteoclast-like phenotypes and molecular markers are evident in BAL and in whole lung of silica-challenged mice. (**A**) TRAP and (**B**) CTSK staining of human silicotic lung tissue (see figs. S7 to S9). Arrows indicate TRAP and CTSK positive macrophage adjacent to fibrotic regions. (**C**) Mouse lung tissue was stained for TRAP (top) and CTSK (bottom) at d0, d28, and d56. (**D**) BAL MNGCs collected at d28 stained with Giemsa, TRAP, and CTSK (see fig. S10). (**E**) TRAP5b, quantified by ELISA, in BALF from saline- or silica-treated mice collected d28 postchallenge (*N* = 4 mice per group). N.D., not detected. (**F**) CTSK activity measured by radiant efficiency of i.t. Cat K 680 FAST delivered d6 postsilica challenge (*N* = 4 mice per group) (see fig. S11). (**G**) RT-qPCR for osteoclast genes in BAL cells (*N* = 4 mice per group) (see fig. S12). (**H**) Scheme for ex vivo osteoclast experiments. (**I**) BAL cells collected at d14 post i.t. challenge with saline (I-1, I-3, and I-5) or silica (I-2, I-4, and I-6). Representative images chosen from cultures supplemented with RANKL (25 ng/ml; see fig. S13) After d6, cells cultured on plastic were stained for TRAP activity (I-1 and I-2; yellow arrows). Cells cultured on bone used phalloidin staining to visualize actin rings (I-3 and I-4; yellow arrows), and resorbed bone area was visualized by peroxidase-conjugated wheat germ agglutinin/horse radish peroxidase staining after removing cells (I-5 and I-6; yellow arrows). Scale bars, 200 μm (I-1 and I-2) and 50 μm (I-3, I-4, I-5, and I-6). Number of actin ring (**J**), actin ring size (**K**), total actin ring area (**L**), and bone resorption area (**M**) were quantified. Error bars show median with interquartile range. *P < 0.05 and **P < 0.01 by Mann-Whitney *U* test or Kruskal-Wallis test followed by Dunn’s test. Scale bars are 20 μm unless noted.

To further validate the presence of osteoclast-like cells identified by both snRNA-seq in the mouse model and scRNA-seq in human patients, we performed immunohistochemistry (IHC) on lung tissue from two patients with silicosis and silica-challenged mice for two signature osteoclast proteins, TRAP and CTSK. TRAP^+^ and CTSK^+^ macrophage and MNGC accumulations were prominent within alveolar spaces surrounding small conducting airways and in focal areas of chronic interstitial inflammation adjacent to fibrotic regions (Fig. 5, A and B). We also stained lung tissue sections from all nine specimens and a donor control used for scRNA-seq studies. Although POLCs were rare in the single-cell dataset, TRAP^+^CTSK^+^ cells are abundant in the lung tissue sections from patients with coal workers pneumoconiosis (CWP; figs. S7 and S8). This staining pattern within end-stage fibrotic lungs provides evidence of durable osteoclast-like differentiation in human silicosis. Similarly, TRAP^+^, CTSK^+^ MNGCs were found in lung tissue and bronchoalveolar lavage (BAL) of silica-treated mice (Fig. 5, C and D), and TRAP^+^ cells can be found in the silica mouse lung 1 year postexposure (fig. S9). MNGCs were present in the BAL of silica-treated mice but not saline-treated controls, representing 4% of recovered cells at d7 and 6% of cells at d28 postsilica treatment (Fig. 5D and fig. S10). TRAP5b, the osteoclast-specific isoform of the acid phosphatase ACP5 (*37*, *38*), was elevated in BAL fluid (BALF) of silica-challenged mice but was not detected in saline control mice at d28 postinstillation (Fig. 5E). Pulmonary CTSK enzymatic activity was evaluated in vivo by i.t. administration of the CTSK cleavage–activated probe, Cat K 680, in mice that had received silica or saline d6 prior. Pulmonary CSTK activity was significantly increased in silica-treated mice compared to saline-treated controls (*P* < 0.05; Fig. 5F and fig. S11).

Next, we sought to validate osteoclastic activity in silica-treated mouse lungs by quantifying RNA levels of multiple osteoclast marker genes using quantitative reverse transcription polymerase chain reaction (RT-qPCR). Silica treatment increased the mRNA levels of *Acp5*, *Ctsk*, *Mmp9*, *Itgb3*, *Atp6v0d2*, *Tnfrsf11a*, *Csf1*, and *Csf1r* in both BAL cells (Fig. 5G and fig. S12A) and whole lung tissue (fig. S12B). For most osteoclast genes, up-regulation occurred by day 7 and plateaued or continued to rise through d28 or d56, suggesting that osteoclast differentiation is likely an early and sustained event in silica-induced lung injury.

To examine capacity for bone resorption, the signature function of osteoclasts, BAL cells collected from saline- or silica-treated mice were cultured with variable concentrations of RANKL (0 to 100 ng/ml) (Fig. 5H). Compared to controls (Fig. 5, I-1, I-3, and I-5), silica challenge enhanced RANKL dose-dependent formation of TRAP^+^ MNGCs, actin rings, and bone pits by isolated BAL cells (Fig. 5, I-2, I-4, and I-6, and fig. S13). Silica-challenged cells demonstrated statistically higher numbers with consistently larger surface area of actin rings compared to controls, and this effect was enhanced when supplemented with exogenous RANKL (Fig. 5, J to L). Bone resorption area increased more markedly with modest RANKL supplementation in silica-treated cells than control cells (Fig. 5M). BAL cells from silica-exposed mice also increased bone matrix proteolytic activity, based on higher media levels of C-terminal telopeptides of type I collagen (CTX-I) when plated on bone slices, compared to BAL cells from saline-exposed mice (fig. S14). Collectively, these results indicate that i.t. silica challenge of mice induces pulmonary myeloid cells to undergo osteoclastic differentiation as indicated by expression of signature osteoclast genes and proteins, formation of TRAP^+^CTSK^+^ MNGCs, actin ring assembly, and acquisition of proteolytic and osteolytic functions.

### Silica-induced RANKL expression activates osteoclast-like cell formation in the lung

**Fig. 6.**
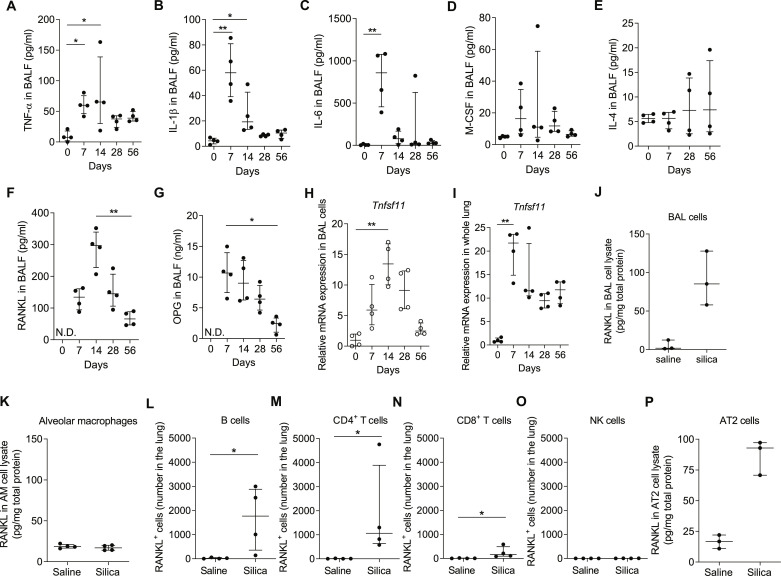
Osteoclast differentiation and activation are RANKL dependent. Analysis of BAL cytokines and cell populations in silica-challenged mice (5 mg, i.t.). TNF-α (**A**), IL-1β (**B**), IL-6 (**C**), M-CSF (**D**), IL-4 (**E**), RANKL (**F**), and OPG (**G**) concentrations, measured by ELISA in BALF at indicated time points (*N* = 4 mice per group). RANKL gene expression, measured by RT-qPCR, in BAL (**H**) and whole lung homogenates (**I**) (*N* = 4 mice per group). RANKL protein concentration, measured by ELISA, in BAL (**J**) and AM (**K**) lysates from silica-treated mice on d14 postchallenge (*N* = 3 to 4 mice per group). Number of RANKL-positive and IFN-γ–negative cells in (**L**) B cells, (**M**) CD4^+^ T cells, (**N**) CD8^+^ T cells, and (**O**) NK cells from whole lungs collected d14 postchallenge (*N* = 4 mice per group) (see fig. S16). (**P**) RANKL in AT2 cell lysates from silica-treated mice on d14 postchallenge, measured by ELISA (*N* = 3 mice per group). Error bars show median with interquartile range. *P < 0.05, **P < 0.01, and ***P < 0.001 by Mann-Whitney *U* test in two groups comparison and Kruskal-Wallis test followed by Dunn’s test in multiple comparisons.

Osteoclastogenesis is differentially regulated by cytokines (*17*, *18*). TNF-α, IL-1β, IL-6, and M-CSF stimulate osteoclast formation and function when even small amounts of RANKL are present (*17*, *18*), while IL-4 suppresses osteoclast formation. Osteoprotegerin (OPG; Tnfrsf11B) acts as a soluble decoy receptor for RANKL (*13*, *14*, *16*), and the ratio of RANKL to OPG determines the level of free RANKL available for osteoclastogenesis (*14*, *39*, *40*). We found that the BALF concentrations of TNF-α, IL-1β, IL-6, and M-CSF but not IL-4 were transiently elevated by silica i.t. treatment (Fig. 6, A to E). Silica increased RANKL and OPG levels in BALF (Fig. 6, F and G) in a staggered temporal pattern in which OPG concentrations peaked on d7, and RANKL peaked on d14 postchallenge. These data indicate that the ratio of RANKL/OPG varied over time in the silica-challenged mouse lung, consistent with time-dependent differential regulation of osteoclastogenesis in the lungs of C57BL/6J mice. The fact that Tnfsf11 gene expression remains elevated in whole lung tissue through at least d56 suggests a mechanism for sustained or successive osteoclast formation.

Osteoblasts and the stromal cells that they are derived from are the classical sources of RANKL in bone (*41*), but the origin of RANKL in the lung is unknown. We found that silica treatment up-regulated RANKL gene expression in both BAL cells and whole lung homogenates (Fig. 6, H and I, and fig. S15). Silica treatment increased RANKL protein expression in BAL cell lysate but not in the purified AM lysate (Fig. 6, J and K), implicating other inflammatory cell sources in the alveolar compartment. Activated lymphocytes have been reported to be a source of RANKL in inflammatory responses (*42*, *43*), and silica exposure increased the number of lymphocytes in BALF (fig. S10). Therefore, RANKL expression in pulmonary lymphocytes was assessed by flow cytometry. Silica exposure induced low, but detectable, cell surface and intracellular RANKL expression in B cells, CD4^+^ T cells, and CD8^+^ T cells but not in natural killer (NK) cells (Fig. 6, L to O), and RANKL expression was induced by silica challenge in multiple pulmonary lymphocyte populations assessed by snRNA-seq (fig. S16). AT2 cells are known to secrete various cytokines (*44*, *45*), and we postulated that they may also secrete RANKL. RANKL protein expression was increased in cell lysates of AT2 cells from d14 silica-challenged mice compared to d14 saline-challenged mice (Fig. 6P), and RANKL mRNA expression was increased in isolated, cultured rat AT2 cells but not bone marrow–derived macrophages exposed to silica ex vivo (fig. S17). Together, these data indicate that pulmonary lymphocytes and AT2 cells are the most likely sources of RANKL in response to silica challenge.

### Silica-induced osteoclast-like differentiation is RANKL dependent

**Fig. 7.**
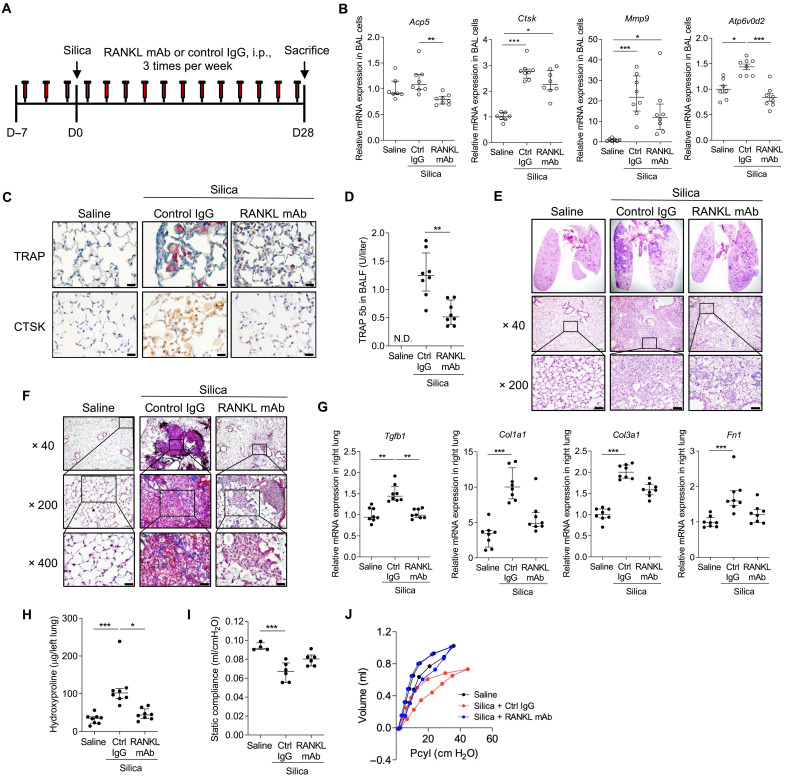
Inhibiting the RANKL-dependent differentiation of POLCs ameliorates pulmonary fibrosis in the silicosis mouse model. (**A**) Study design for anti-RANKL mAb studies. Mice treated with RANKL mAb or Ctrl IgG (0.25 mg per mouse, i.p., three times per week) were challenged with silica (5 mg) and euthanized d28 later. (**B**) RT-qPCR for osteoclast-related genes: *Acp5* (TRAP), *Ctsk*, *Mmp9*, and *Atp6v0d2* in BAL cells (*N* = 7 to 9 mice per group) (see fig. S18). (**C**) Lung sections were stained for TRAP enzymatic activity (top) or with anti-CTSK (bottom) (*N* = 6 mice per group) (see fig. S19). Scale bars, 20 μm. (**D**) TRAP5b in BALF, measured by ELISA (*N* = 7 to 9 mice per group). Paraffin-embedded lung sections were stained with (**E**) H&E and (**F**) Masson’s trichrome reagent (*N* = 6 mice per group). Scale bars, 20 μm. (**G**) Expression of fibrosis-related genes quantified in right lungs using RT-qPCR (*N* = 7 to 9 mice per group). (**H**) Hydroxyproline levels in left lungs were quantified (*N* = 7 to 9 mice per group). Pulmonary function tests performed included static compliance (**I**) and *P*-*V* curve analysis (**J**) (see fig. S20) (*N* = 4 to 6 mice per group). Error bars show median with interquartile range. *P < 0.05, **P < 0.01, and ***P < 0.001 by Mann-Whitney *U* test in two groups comparison and Kruskal-Wallis test followed by Dunn’s test in multiple comparisons.

To examine the role of RANKL in osteoclast-like differentiation in the silica-challenged lung, we investigated whether a neutralizing anti-mouse RANKL monoclonal antibody (mAb; clone, IK22-5) (*15*, *46*–*49*) attenuates silica-induced osteoclastogenic programs (Fig. 7A). Anti-RANKL mAb treatment delivered by the intraperitoneal (i.p.) route three times weekly suppressed silica-induced expression of osteoclast-related genes *Acp5*, *Ctsk*, *Mmp9*, and *Atp6v0d2* in both BAL cells (Fig. 7B) and in whole lung homogenates (fig. S18) compared to control (Ctrl) immunoglobulin G (IgG). Anti-RANKL also reduced TRAP enzymatic activity and CTSK expression in tissues sections compared to Ctrl IgG, especially within MNGCs (Fig. 7C). Semiquantitative analysis of brown pixel density confirmed a decrease in CTSK staining in silica-challenged mice treated with anti-RANKL, although no difference was detected in red pixel density for TRAP staining at d28 (fig. S19). However, anti-RANKL mAb treatment significantly decreased the levels of TRAP5b in BALF compared to Ctrl IgG as measured by enzyme-linked immunosorbent assay (ELISA) (Fig. 7D). Collectively, these data indicate that silica treatment activates osteoclast-like differentiation in the lung in a RANKL-dependent manner.

### Anti-RANKL mAb suppresses silica-induced pulmonary fibrosis

We postulated that tissue injury from osteoclast-derived matrix degrading proteases and hydrochloric acid are key drivers of progressive lung remodeling in silicosis. Multiple histologic, immunohistochemical, and physiologic approaches were applied to determine whether anti-RANKL mAb–induced inhibition of osteoclastogenesis attenuates silica-induced pulmonary fibrosis. Tissue staining revealed that silica-dependent pulmonary inflammation and fibrosis were significantly attenuated by 5 weeks of anti-RANKL antibody treatment as compared to Ctrl IgG treatment based on Ashcroft scoring and examination of tissue sections (Fig. 7, E and F, and fig. S20). Fibrotic gene expression and the accumulation of hydroxyproline in lung tissues decreased with anti-RANKL mAb treatment (Fig. 7, G and H). In addition, the *P*-*V* curve of silica-treated mice receiving Ctrl IgG was shifted down and to the right compared with saline controls, consistent with reduced compliance, whereas the position of the *P*-*V* curve of silica-treated animals receiving anti-RANKL mAb partially corrected toward the vehicle control, consistent with partial rescue of silica-related reduction in compliance (Fig. 7, I and J). These data demonstrate that inhibiting osteoclastic activation via anti-RANKL reduces overall fibrotic burden, implicating POLCs in the pathogenesis of silica induced pulmonary fibrosis.

## DISCUSSION

Silicosis is an intractable and inexorably progressive form of pulmonary fibrosis that remains a global occupational threat. We used high-throughput genomic methods including scRNA-seq on fibrotic human lungs with known silica exposure and snRNA-seq on whole lung samples from a longitudinal study of silicotic mice to explore novel mechanisms of silica-induced fibrosis. These methods uncovered distinct cell states that temporally contribute to inflammation and fibrosis in silicosis. Activation of an osteoclast genetic program occurs in both tissue-resident and recruited monocytes, indicating that this shift is lineage independent and driven by tissue-specific microenvironmental factors. We validated the presence of osteoclast-like cells and osteoclast differentiation in human and mouse lung tissues using qRT-PCR and IHC and in mouse models by biomarker analysis and osteoclast functional assays (multinucleation, bone pitting, actin ring formation, and TRAP and CTSK activity). We determined that silica challenge induced expression of the signature osteoclastogenic cytokine, RANKL, and identified the lung cell compartments that it was likely derived from, lymphocytes (*42*, *43*) and AT2 cells. We believe that monocytes gain an osteoclast-like phenotype after arriving in the lung and adopting a more polarized macrophage phenotype (for example, Infl-2 and Fibr-2). Collectively, these data suggest aberrant induction of osteoclastic gene program in silica-exposed lung macrophages drives a pathogenic response to lung injury.

Because silica particles are nondissolvable and remain in the lung indefinitely, they may promote a self-perpetuating program of constitutive differentiation of POLCs as the particles are taken up by phagocytes and released again after cell death. Although the life span of bone osteoclasts is typically measured in weeks (*50*, *51*), POLCs are found in the end-stage lung of patients with silicosis decades after exposure and in the silica mouse lung for at least 1 year after i.t. challenge (fig. S9). Our longitudinal data suggest that RANKL is a persistent major osteoclastogenic stimulus in the lung following silica challenge (Fig. 6, H and I). Disruption of the RANKL/RANK axis with a mAb attenuated osteoclastogenesis and the histological, immunohistochemical, genetic, biochemical, and physiological hallmarks pulmonary fibrosis, suggesting RANKL as a promising target for future disease intervention.

Osteoclastogenesis has rarely been reported in compartments other than bone, and it remains unclear whether the POLCs in the silicosis model represent the fully differentiated osteoclasts found in bone. Yet, these ectopic cells demonstrate the most specific distinguishing physical features of true osteoclasts including formation of actin rings and the capacity to degrade the mineral and matrix components of bone. Whether better described as osteoclasts or osteoclast-like, these cells are clearly differentiating along an osteoclast pathway at both molecular and functional levels and represent an interesting new pulmonary myeloid cell phenotype of potential pathophysiologic significance in silicosis.

One important limitation in this study was the small number of osteoclast-like cells identified in the human single-cell data despite the finding that TRAP^+^CTSK^+^ cells were quite prevalent in the human CWP lungs by IHC (figs. S7 and S8). It should be noted that, however, MNGC would be expected to be underrepresented in single-cell samples compared to single-nuclear samples, because of difficulties encountered with dissociation of viable cells from the matrix and exclusion of large cell aggregates from the channel during isolation. In addition, it may well be that osteoclast-like cells are less abundant in end-stage lungs and that sampling error could result in variable capture in a disease where fibrosis is nonuniform. Last, although silica exposure levels can certainly vary in miners based on their working proximity to the coal face and the silica content of the coal dust inhaled, Appalachia, where our coal workers were from, has the highest respirable silica levels in the nation (*52*).

In summary, we find that pulmonary silica exposure incites progressive fibrosis in part via RANKL-dependent osteoclastic transformation of pulmonary myeloid cells, likely through their sustained elaboration of matrix degrading proteases and hydrochloric acid. These findings showcase the utility of single-cell genomic profiling of whole tissue combined with multiple modes of biological validation to identify both a novel mechanism of pulmonary fibrosis and a promising new therapeutic target for silicosis, the POLC. Although osteoclastic transformation of monocytes is a known component of bone homeostasis in adult organisms, our study demonstrates that activation of a routine genetic program in an atypical location can produce a pathological result. This study illustrates how collecting molecular measurements of all genes in all cell types at once in an authentic disease model is a powerful approach to understanding complex pathobiology and to identifying new molecular targets for treatment of the disease.

## MATERIALS AND METHODS

### Experimental design

Silicosis is a life-threatening, progressive lung disease limited by a poor understanding of pathophysiology and a lack of effective treatments. We explored mechanisms of silica-induced pulmonary fibrosis in human lung samples collected from patients with occupational exposure to silica and in a longitudinal mouse model of silicosis using multiple modalities including whole-lung scRNA-seq and histological, biochemical, and physiologic assessments. Silica particles were administered via the i.t. route into the lungs of C57BL/6 mice. Lung fibrosis in tissues from patients with silicosis and silica-challenged mice was characterized, using fibrosis-related gene expression quantified by RT-qPCR, hydroxyproline measured in lung homogenates, and lung compliance measured using oscillatory impedance. To explore novel mechanisms of silica-induced fibrosis, scRNA-seq and snRNA-seq were conducted on tissues from patients with silicosis and silica-challenged mice, respectively. To validate these findings, the presence of osteoclast-like cells and osteoclast differentiation in human and mouse lung tissues was interrogated with RT-qPCR, IHC, biomarker analysis, and osteoclast functional assays. Cytokines known to participate in osteoclast differentiation were measured in BALF by ELISA. The finding that silica exposure induced regional expression of the signature osteoclastogenic cytokine, RANKL, led to a search for the source. Increased RANKL protein expression was identified after silica challenge in isolated AT2 by ELISA and in lymphocytes by ELISA and flow cytometry. The osteoclastogenic propensity of cultured BAL cells isolated from mice before and after silica challenge was tested by determining the threshold of RANKL concentration required for robust, multinucleated osteoclast formation. Fibrosis assessed silica-challenged mice treated with anti-RANKL by the intraperitoneal route using histology, RT-qPCR, hydroxyproline assay, and pulmonary function testing. Because the silica-challenged animals were easily distinguished from controls based on the presence of particles in lung tissue, randomization and blinding were not used for experiments with animals, but mice were age- and sex-matched for all studies. The experimental procedures were approved by the Institutional Animal Care and Use Committee at the University of Cincinnati.

### Histology

Archived, anonymized paraffin-embedded surgical lung specimens from human patients with silicosis were provided by C. Shaver, M.D., Ph.D and J. Kropski, M.D. at Vanderbilt University Medical Center (Nashville, TN) (Institutional Review Board #060165 and #192004). Mouse lung tissues were fixed with 10% buffered formalin phosphate, embedded in paraffin, and stained with H&E. Masson’s trichrome staining was used to identify collagen deposition (Newcomer Supply, Middleton, WI).

#### Animals

Sprague-Dawley rats used for alveolar type II cell isolation were obtained from Charles River Laboratories (Wilmington, MA). C57BL/6J mice were obtained from the Jackson Laboratory (Bar Harbor, ME). All mice used in experiments were male, 8 to 10 weeks old, and 23 to 27 g in weight. The dosing strategy used was 5-mg instilled intratracheally into 25 g of mice or ~200 mg/kg body weight, unless otherwise indicated. For terminal experiments, mice or rats were euthanized by intraperitoneal injection of Euthasol (Henry Schein, Melville, NY). Chow that contained 0.7% phosphate, 16.3% protein, 66.3% carbohydrate, 5.0% fat, 1.2% calcium, and vitamin D3 (2900 IU/kg) was prepared by Harlan Laboratories (Madison, WI) and purchased from Harlan Sprague Dawley (Indianapolis, IN, USA). All animals were maintained in a specific pathogen–free facility and were handled according to a University of Cincinnati Institutional Animal Care and Use Committee approved protocol and National Institutes of Health guidelines.

### In vivo exposure to silica

Silica particles (Sigma-Aldrich, St. Louis, MO, catalog number S5631; particle size: 80% between 1 and 5 μm, 99% between 0.5 and 10 μm) were boiled in 1 N HCl for 1 hour, washed with deionized H_2_O, and dried at 100°C. The particles were then heat sterilized at 200°C for 2 hours and suspended in sterile saline. The endotoxin content of the silica particles was silica (<1.0 pg/μg) as determined using the LAL Chromogenic Endotoxin Quantitation Kit (Thermo Fisher Scientific, Rockford, IL) according to the manufacturer’s instructions. For i.t. silica administration, C57BL/6J mice were anesthetized with isoflurane and suspended by their incisors in the supine position on a procedure board at a 45° angle. The glottis was visualized by retraction of the tongue and illuminated with a fiberoptic thread. A 22-gauge angiocatheter was advanced into the trachea under direct visualization, and after confirming correct placement by expansion of the thorax upon delivery of air through the catheter, 5 mg of silica in saline was injected into the lung. For oropharyngeal (o.a.) silica administration, each mouse was anesthetized with isoflurane and suspended by a steel wire on a procedure board at a 60°C angle by the incisor teeth. The mouth was opened, the tongue was pulled forward, and 5 mg of silica in saline was placed at the base of the tongue. Once the slurry was aspirated into the lungs with inspiration, the tongue was released.

### Preparation of RNA and RT-qPCR

Total RNA was isolated from the murine BAL pellet and murine whole lung tissues using RNAzol RT (Molecular Research Center, Cincinnati, OH), according to the manufacturer’s instructions. cDNA was synthesized using the High-Capacity cDNA Reverse Transcription Kit (Applied Biosystems, Waltham, MA). RT-qPCR was performed using a SYBR Green Master Mix (Applied Biosystems) with primer pairs for fibrosis-related and osteoclast-related genes, as well as β-actin and actin α-2 smooth muscle as internal controls. The nucleotide sequences of the primer pairs are included in table S3.

### Measurement of hydroxyproline content in the mouse lung

For hydroxyproline measurements, lung tissues were homogenized with 1 ml of deionized H_2_O per 100 mg of tissue. Equal volumes of 12 M HCl and lung homogenate were mixed, and the samples were hydrolyzed at 120°C for 3 hours and centrifuged at 10,000*g* for 3 min. The supernatant was collected, and hydroxyproline in the supernatant was determined using the Hydroxyproline Assay Kit (Sigma-Aldrich), according to the manufacturer’s instructions.

### Lung physiological measurements using SCIREQ Flexivent System

C57BL/6J mice were anesthetized with isoflurane or ketamine and intubated through a tracheostomy with a metallic angiocatheter. Lung compliance and pressure volume characteristics were measured using oscillatory impedance (Flexivent, version 5.1, SCIREQ Scientific Respiratory Equipment Inc., Montreal, Canada) and plotted using GraphPad Prism (ver. 9.5.1., GraphPad Software, San Diego, CA) as previously reported (*25*, *53*).

### Human lung single-cell sample collection and library generation

Fibrotic lung tissue samples were obtained from individuals with CWP at the time of lung transplantation. Nonfibrotic control tissue samples were obtained from donor lungs that were declined for organ donation. For CWP and control lungs, tissue sections were taken from within ~2 cm of the pleural surface. For five of the CWP donors, lung sections from more and less fibrotic areas were collected, based on preoperative chest computed tomography. For control lungs, the most normal-appearing region was identified by gross inspection and selected for biopsy. Single-cell suspensions were generated using a collagenase/dispase enzymatic cocktail and serially filtered as previously described. CD45^+^ and CD45^−^ cells were sorted using magnetic beads (Miltenyi Biotec) and mixed at a 1:2 ratio for library preparation. scRNA-seq was performed using the 10× chromium platform with 5′ library preparation kits and was sequenced on an Illumina NovaSeq 6000 as previously reported (*54*).

### Mouse lung dissociation and nuclei fixation

Snap-frozen mouse lung tissue (93 to 223 mg) was dissociated using a gentleMACS tissue dissociator using “C” dissociation tubes in 5 ml of ice-cold lysis/fixation buffer [10 mM NaCl, 10 mM sodium phosphate (pH 7.2), 3 mM MgCl_2_, 5% glutaraldehyde, 10 mM vanadyl ribonucleoside complex, 0.1% Triton X-100, 1% diethyl pyrocarbonate, and 0.00015% polyvinyl sulfonic acid (Sigma-Aldrich, catalog no. 278424)]. Tissue was dissociated using the “Mouse Spleen 1” program (gentleMACS dissociator, Miltenyi Biotec) for 60 s and then filtered using a 70-μm cell strainer. The strainer was washed with an additional 5 ml of lysis/fixation buffer, and then nuclei were fixed at 4°C for 15 min and pelleted by centrifugation at 500 relative centrifugal force (RCF), 4°C, for 8 min. The supernatant was discarded, and nuclei were resuspended in 1 ml of nuclei suspension buffer [10 mM tris HCl (pH 7.4), 10 mM NaCl, and 3 mM MgCl_2_]. Nuclei were filtered through a 30-μm strainer and then pelleted by centrifugation at 500 RCF for 5 min at 4°C. The supernatant was discarded, and nuclei were resuspended in 500 μl of nuclei suspension buffer and pelleted again at 500 RCF at 4°C for 5 min. After discarding the supernatant, the nuclei pellet was resuspended in 210 μl of nuclei suspension buffer. Two aliquots of 100 μl of nuclei suspension were snap-frozen in liquid nitrogen and stored in liquid nitrogen for single-cell combinatorial indexing RNA sequencing (sci RNA-seq) library preparation.

### Sci RNA-seq library generation

snRNA-seq data were prepared using the two-level workflow for sci RNA-seq (*55*). The protocol was modified to use the following room temperature incubation temperatures, instead of a 55°C /5 min of incubation: 2 min at each of 4°, 10°, 20°, 30°, 40°, 50°C followed by 10 min at 53°C and 15 min at 55°C. Samples were prepared using 6 plates of room temperature indices (for 576 indices used) divided evenly between the 12 samples. One hundred fifty nuclei in total were sorted into each well of the four PCR plates that were prepared. Libraries were sequenced using an Illumina Nextseq 550.

### Sequencing data processing and analysis

Raw sequencing output was processed as previously described (*56*), and analysis was performed using Monocle 3 (v1.2.9) (*57*). Cells were filtered for quality control based on the following thresholds: >100 unique molecular identifiers (UMIs), <10% mitochondrial RNA, and <0.2 Scrublet (*58*) doublet score. For visualization, we performed dimensionality reduction using the first 100 principal components, followed by two-dimensional Uniform Manifold Approximation Projection (UMAP) projection. We hierarchically annotated the data (into broad and fine cell states) using Louvain clustering with varying levels of resolution in combination with marker genes identified from literature.

### Differential abundance testing

Cell numbers were collapsed per sample according to the fine cell state annotation. Cell numbers were size factor–normalized to correct for different cell numbers recovered from samples. A beta-binomial test with Benjamini-Hochberg correction for multiple hypotheses was used to test for differences in cell state abundance for each time point compared to baseline (d0). A corrected *P* value less than 0.05 was considered significant.

### Gene signature analysis

Gene sets were acquired from Molecular Signatures Database (MSigDB) (*36*) (Hallmarks Inflammation Gene Set, GO_Osteoclast_differentiation), Aran *et al.* (*59*) (M1/M2 signatures), and Wang *et al.* (*60*) (fibrosis signature). For each gene, we translated the human gene set to mouse orthologs using the gorth function from the gprofiler2 package in R. For M1 and M2 analyses, only genes unique to one of the lists were used. A summary score of gene set activation was calculated per cell using the aggregate_gene_expression function in Monocle3 (*57*). Scores were calculated using log-transformed expression values and were normalized to a scale of −3 to 3.

### Differential expression analysis

To test for differences in gene expression over time in the sequencing experiment, we used a linear mixed effect model with splines. Significant differentially expressed genes were annotated with GO function terms using g:Profiler. Terms with *P* < 0.05 corrected for multiple hypothesis testing with the default g:SCS method were considered significant.

### Pseudotime analysis

We calculated pseudotime for the IMs to capture pathway activation changes associated with macrophage polarization and plasticity. Pseudotime was calculated using learn_graph in Monocle3 (*57*) with default parameters except for use_partitions = FALSE. The root node was designated on the basis of end with the highest number of cells collected from d0. Genes, expressed in at least 100 cells, that vary along the pseudotime trajectory were identified using linear model with natural splines (df = 3). Differentially expressed genes were selected on the basis of *q*_value < 0.05. Significant differentially expressed genes were annotated with GO function terms using g:Profiler. Terms with *P* < 0.05 corrected for multiple hypothesis testing with the default g:SCS method were considered significant.

### IHC and immunocytochemistry

Immunohistochemical analysis was performed on formalin-fixed, paraffin-embedded lung specimens. Tissue sections were deparaffinized, rehydrated with deionized H_2_O, subjected to antigen retrieval with citrate buffer, and blocked with normal serum. For TRAP staining, tissue sections were incubated with prewarmed (37°C) TRAP staining mix [50 mM MES (pH 4.8) containing 50 mM l-(+) tartaric acid, 0.3 mM naphthol AS-MX phosphate, and 1.5 mM Fast Red Violet LB salt (Sigma-Aldrich)] for 15 min and counterstained with hematoxylin for 5 s. For immunocytochemistry, cells were cytospun onto glass slides (700 rpm, 5 min), dried overnight, fixed with 3.7% neutral-buffered formalin. Specimens were incubated with anti-CTSK antibody (polyclonal, 1:750) obtained from Abcam (Cambridge, UK), followed by horseradish peroxidase–linked anti-rabbit secondary antibody (Cell Signaling Technology, Beverly, MA). The 3-3′-Diaminobenzidine (DAB) substrate kit (Thermo Fisher Scientific) was used for color development, and samples were counterstained with hematoxylin for 5 s. Semiquantitative analysis was performed using ImageJ.

### BAL cells and fluid collection

Mice were euthanized, and tracheostomy was performed. A plastic cannula was inserted through the incision, and the lung was flushed with 1 ml of saline and repeated five times. The total volume recovered was approximately 4 ml per mouse. The BALF was centrifuged at 500*g* for 10 min to pellet the cells. Gene expression in BAL cells was assessed by RT-qPCR, and the cytokines in the supernatant from the first lavage cycle were quantified by ELISA.

### Cytology

For cytology, BAL cells were spun onto glass slides (700 rpm, 5 min), dried overnight, and incubated with Giemsa solution (Sigma-Aldrich) for 1 min and washed with deionized H_2_O for 5 min, three times.

### Protein sample preparation

A total of 2.0 × 10^7^ of BAL cells, isolated AM, or isolated AT2 cells were lysed by suspension in 1 ml of lysis buffer containing protease and phosphatase inhibitors (radioimmunoprecipitation assay lysis buffer system, Santa Cruz Biotechnology, Santa Cruz, CA) for 30 min on ice and centrifuged at 14,000*g* for 10 min, and the supernatant was collected. The concentrations of protein samples were measured using the Pierce BCA Protein Assay Kit (Thermo Fisher Scientific). The concentrations of RANKL in BAL cell lysate, AM lysate, or AT2 cell lysate were measured by ELISA and normalized to total protein concentrations.

### Enzyme-linked immunosorbent assay

The levels of analytes in mouse BALF were measured by ELISA as follows: TRAP 5b, Mouse TRAP (TRAP 5b) ELISA Kit (Immunodiagnostic Systems, UK); TNF-α, Mouse TNF-α Uncoated ELISA Kit (Invitrogen, Waltham, MA); IL-1β, Mouse IL-1β Uncoated ELISA Kit (Invitrogen, Minneapolis, MN); IL-6, Mouse IL-6 DuoSet ELISA Kit (R&D Systems Inc.); IL-4, Mouse IL-4 DuoSet ELISA Kit (R&D Systems Inc.); M-CSF, Mouse M-CSF DuoSet ELISA Kit (R&D Systems Inc.); and OPG, Mouse OPG/TNFRSF11B DuoSet ELISA Kit (R&D Systems Inc.) according to the manufacturer’s instructions. The levels of RANKL in mouse BALF and AT2 and BAL cell lysates were measured using the Mouse TRANCE/RANK L/TNFSF11 Quantikine ELISA Kit (R&D Systems Inc.).

### Fluorescent live imaging

Cat K 680 FAST Fluorescent Imaging Agent (PerkinElmer Inc., Hopkinton, MA) was used to in vivo detect CTSK activity in the lung. C57BL/6J mice that had been intratracheally challenged with silica or saline 1 week prior were shaved around the chest before imaging, and 1 nmol of Cat K 680 FAST probe was administered by the i.t. route. The fluorescence signal emanating from the chest was monitored using fluorescent live imaging system (IVIS Spectrum, PerkinElmer Inc.) at 18 hours postinjection, and fluorescence intensity was analyzed as previously described (*61*, *62*).

### Osteoclast formation, bone resorption pit assay, and actin ring assembly

BAL cells were collected d14 after i.t. saline or silica challenge and plated on plastic or bovine bone slices (Immunodiagnostic Systems, UK) in 96-well plates (2.0 × 10^4^ cells per well) in α-minimum essential medium (Gibco) containing 10% fetal bovine serum (FBS) in the presence of various concentrations (0 to 100 ng/ml) of recombinant murine RANKL (PeproTech) and 1:40 dilution of CMG12-14 [murine M-CSF–producing cell line (*63*)] conditioned medium (equivalent to 30 ng/ml of recombinant human M-CSF). Medium was changed on d2, d4, and d5. Cells plated on plastic were fixed in formalin and stained for TRAP activity after d6 in culture. For actin ring staining, cells on bone slices were fixed on d6 in formalin and permeabilized in 1% Triton X-100, rinsed with phosphate-buffered saline (PBS), and stained with Alexa Fluor 488–phalloidin (Invitrogen) (*64*). For the bone pitting assay, cells on bone slices were removed by scrubbing the bone surface with a toothbrush, and resorption pits were visualized by incubation with peroxidase-conjugated wheat germ agglutinin (20 μg/ml) and staining with 3,3′-diaminobenzidine (Sigma-Aldrich) (*63*, *64*). The levels of CTX-I in culture medium over bone slices were measured using the CrossLaps for culture (CTX-I) ELISA (Immunodiagnostic Systems) (*65*).

### AM collection in mouse

C57BL/6J mouse lungs were euthanized at the indicated times after i.t. saline or silica challenge, and BAL cells were collected as above. Cell pellets were resuspended in Dulbecco’s modified Eagle’s medium (DMEM) with 10% fetal calf serum (FCS) and plated for isolation of AM by adherence to tissue culture plastic.

### Isolation of mouse and rat AT2 cells

C57BL/6J mouse lungs were harvested on d14 after i.t. saline or silica challenge. Lungs were perfused with sterile normal saline via the pulmonary artery. The airway was cannulated via tracheostomy with an angiocatheter, and 2 ml of dispase (50 U/ml; Corning Inc., Corning, NY) was instilled, followed by 0.5 ml of 1% low-melt agarose (warmed to 45°C). Lungs were rapidly cooled on ice for 2 min, submerged in dispase for 45 min at room temperature, and transferred to a culture dish containing deoxyribonuclease I (DNase I; 0.1 mg/ml; Sigma-Aldrich). The parenchymal lung tissue was gently teased from the bronchi and homogenized. Cell suspensions were filtered, collected by centrifugation, and panned over prewashed 10-cm tissue culture plates coated with CD45 and CD32/16 antibodies (BD Biosciences, San Jose, CA). After incubation for 60 min at 37°C in a 5% CO_2_ atmosphere to promote adherence of contaminating macrophages and fibroblasts, the AT2 cells were gently decanted from the plate, collected by centrifugation, and counted. Rat AT2 were isolated using published methods (*66*, *67*) with modifications. Animals were anesthetized with intraperitoneal sodium pentobarbital (75 mg/kg) and exsanguinated via the abdominal aorta. The right ventricle was cannulated, and the lungs were perfused with heparin and flushed with 10 ml of Hepes-buffered saline (HBS). The lungs were removed, lavaged with HBS + Ca, filled with 33 ml of elastase solution in Hanks’ balanced salt solution (HBSS) + Ca (3 U/ml), and incubated at 37°C for 20 min. The lung parenchyma was cut into 1-mm pieces with scissors and incubated with 425 μl of DNase (Sigma-Aldrich, #D4527; 17 U/ml) in 10 ml of Balanced salt solution B (BSS-B) at 37°C with swirling. The suspension was filtered through two layers of gauze and 100- and 20-μm nylon filters to remove cell clumps and debris. The cells were centrifuged (8 min, 300*g*, 4°C), and the pellet was resuspended in buffer to remove red blood cells and respun. The cell pellet was resuspended in DMEM-HEPES and panned over a rat IgG-coated plate (Sigma-Aldrich, # I4131) for 1 hour at 37°C in a 5% CO_2_ atmosphere to remove macrophages. The supernatant was centrifuged (8 min, 300*g*, 4°C) to collect epithelial cells, and the supernatant was resuspended in 10 ml of DMEM + 10% FBS and 1% penicillin/streptomycin and plated on collagen IV plates (Corning, 354430).

### Lung digestion for flow cytometry

The pulmonary vasculature was flushed with PBS (−) via right ventricular puncture. Lungs were removed and transferred to Iscove’s modified Dulbecco’s medium (Lonza, Malkersville, MD) containing 10% FCS, DNase I (0.1 mg/ml; Sigma-Aldrich), and Liberase TL (0.5 mg/ml; Roche, Basel, Switzerland). Lungs were minced with scissors and incubated at 37°C for 45 min, after which time they cooled on ice and pushed through a 70-μm strainer. Specimens were centrifuged at 1200 rpm at 4°C, resuspended in Red Blood Cell Lysing Buffer (Sigma-Aldrich) for 2 min, washed with media, and filtered through 50-μm mesh. Cell viability was determined by trypan blue exclusion. Cells were plated into 96-well polystyrene round-bottomed culture plates at a density of 1 × 10^6^ cells per well in preparation for stimulation and staining.

### Flow cytometry

For intracellular staining, the cells were treated with eBioscience Cell stimulation cocktail (500×) consisting of phorbol 12-myristate 13-acetate and ionomycin (Invitrogen, Walthman, MA), eBioscience brefeldin A solution (1000×) (Invitrogen), and eBioscience Monensin Solution (1000×) (Invitrogen) at 37°C for 2 hours. The cells with or without stimulation were stained with the Live/Dead Aqua Dead Cell Stain Kit (Invitrogen), BUV395 rat anti-mouse CD45 (BD Biosciences), BV605 rat anti-mouse CD90.2 (BD Biosciences), Allophycocyanin (APC) anti-mouse CD3 (BioLegend, San Diego, CA), phycoerythrin (PE)–Cyanine 7 (Cy7) anti-mouse CD4 (BioLegend), PE-CF594 rat anti-mouse CD8a (BD Biosciences), peridinin-chlorophyll-protein complex (PerCP)–Cyanine5.5 anti-mouse CD19 (BioLegend), fluorescein isothiocyanate anti-mouse NK-1.1 (BioLegend), PE anti-mouse CD254 (TRANCE, RANKL) (BioLegend), and BV421 rat anti-mouse INF-γ (BD Biosciences) at 4°C, overnight. Four staining reactions per mouse were pooled before analyzing on an LSR Fortessa II cytometer (Becton, Dickinson and Company, Franklin Lakes, NJ). Data were analyzed with FlowJo software (TreeStar, Ashland, OR).

### Anti-RANKL mAb treatment

C57BL/6J mice were treated with an anti-mouse RANKL mAb (clone IK22-5, Bio-X-Cell, West Lebanon, NH) or a rat IgG2a isotype control IgG (clone 2A3, Bio-X-Cell) (0.25 mg per mouse, i.p., three times per week) 1 week before i.t. or o.a. challenge with 5 mg of silica particles. After the silica administration, mice continued to be treated with the anti-RANKL mAb or a rat IgG2a isotype IgG three times per week and then euthanized at indicated times postsilica administration, followed by assessments by histology, immunochemistry, and hydroxyproline content of lung tissues and RT-qPCR and osteoclast functions of BAL cells. Some mice underwent pulmonary function testing as described above.

### Data access

We used the genes annotated to “osteoclast differentiation” from GO (downloaded from MSigDB website link: https://gsea-msigdb.org/gsea/msigdb/human/geneset/GOBP_OSTEOCLAST_DIFFERENTIATION.html). Sequencing data was submitted to Gene Expression Omnibus.

### Study approval

Human sample collection and laboratory analysis were approved by the Institutional Review Board at The University of Cincinnati College of Medicine and Vanderbilt University Medical Center, and informed consent was obtained per protocol. The animal experimental procedures were approved by the Institutional Animal Care and Use Committee at the University of Cincinnati.

### Statistical analysis

Statistical analyses were performed using GraphPad Prism (version 9.5.1, GraphPad Software). Differences between two groups were compared using nonparametric Mann-Whitney *U* test. In experiments in which more than two groups were involved, nonparametric Kruskal-Wallis test followed by Dunn’s multiple comparisons test was used. Differences were considered significant at *P* < 0.05.
