# Supplementary material for: Pulmonary osteoclast-like cells in silica induced pulmonary fibrosis
Source: Sci Adv. 2024 Jul 10;10(28):eadl4913. doi: 10.1126/sciadv.adl4913 (PMC11235167; doi:10.1126/sciadv.adl4913)
Supplement: Supplementary file 1 — Figs. S1 to S20 Tables S1 to S4 [file sciadv.adl4913_sm.pdf]

Science Advances
AAAS

Supplementary Materials for

Pulmonary osteoclast-like cells in silica induced pulmonary fibrosis

Yoshihiro Hasegawa *et al.*

Corresponding author: Yoshihiro Hasegawa, y-hasegawa@sapmed.ac.jp; Jennifer M. Franks, jmfranks@uw.edu; Cole Trapnell, coletrap@uw.edu; Francis X. McCormack, frank.mccormack@uc.edu

Sci. Adv. **10**, eadl4913 (2024)
DOI: 10.1126/sciadv.adl4913

This PDF file includes:

Figs. S1 to S20
Tables S1 to S4

Fig. S1, relates to Fig. 2

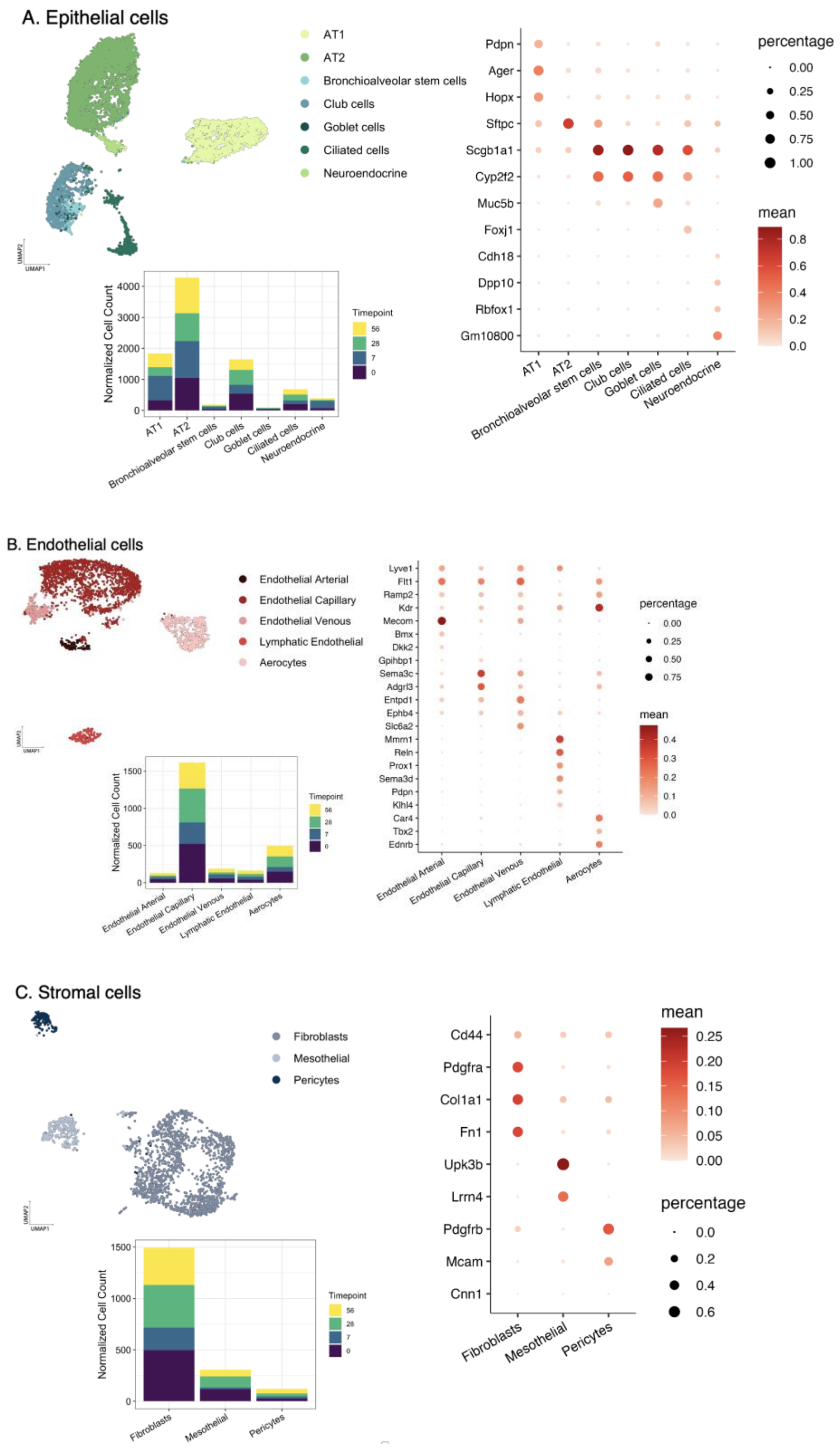

Fig. S1 (cont)

D. Myeloid cells

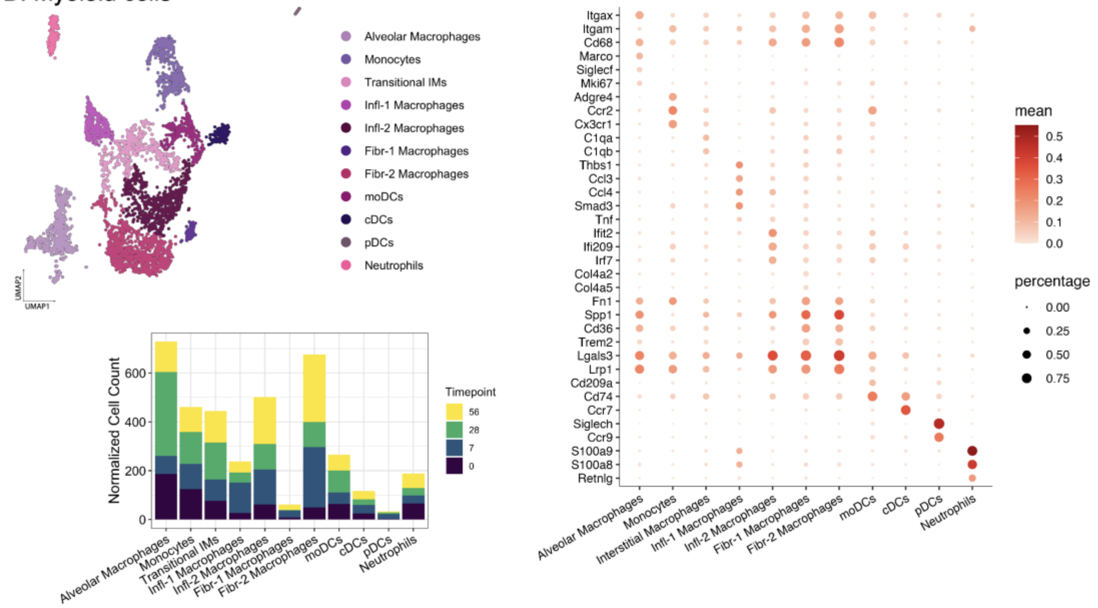

E. Lymphoid cells

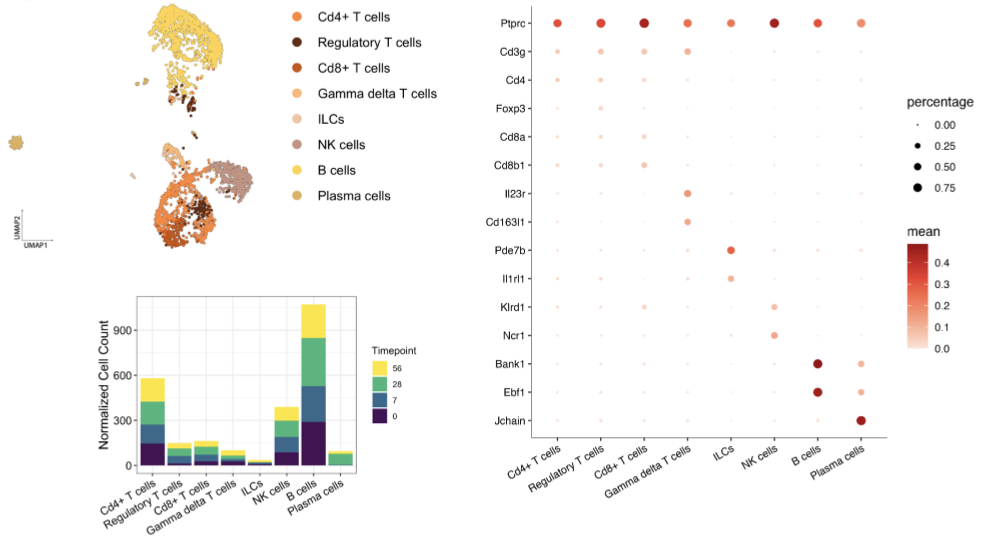

Fig. S1. Fine annotation of single-nucleus RNA-sequencing data from silica mice. RNA from single nuclei were isolated and sequenced from intratracheal silica challenged mice at Day 0 (pre-silica exposure) and Days 7, 28, and 56 (post-silica exposure). Cells were annotated based on clustering in reduced dimensions (left panels) and expression of marker genes (center panels) within major cell lineages in the murine lung: epithelial (A), endothelial (B), stromal (C), myeloid (D), and lymphoid (E). Total normalized number of each cell type are quantified and colored according to timepoint (right panels).

Fig. S2

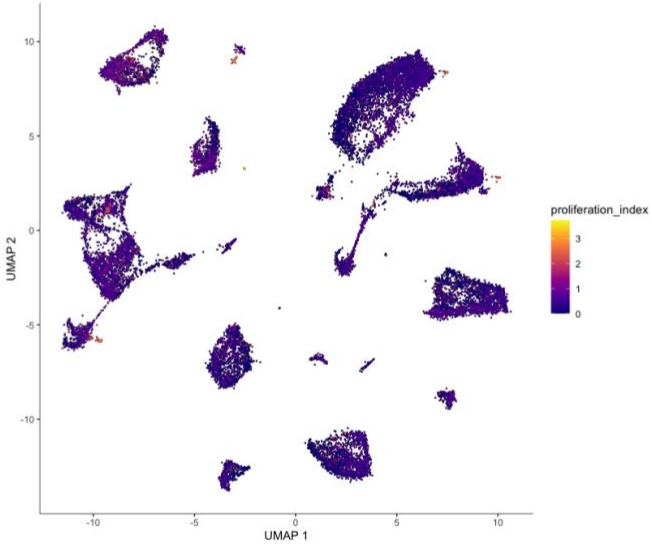

Fig. S2 Proliferation index plotted for silica mouse time-course. Small distinct clusters with consistently high proliferation indices were annotated as cycling cells.

Fig. S3, relates to Fig. 3

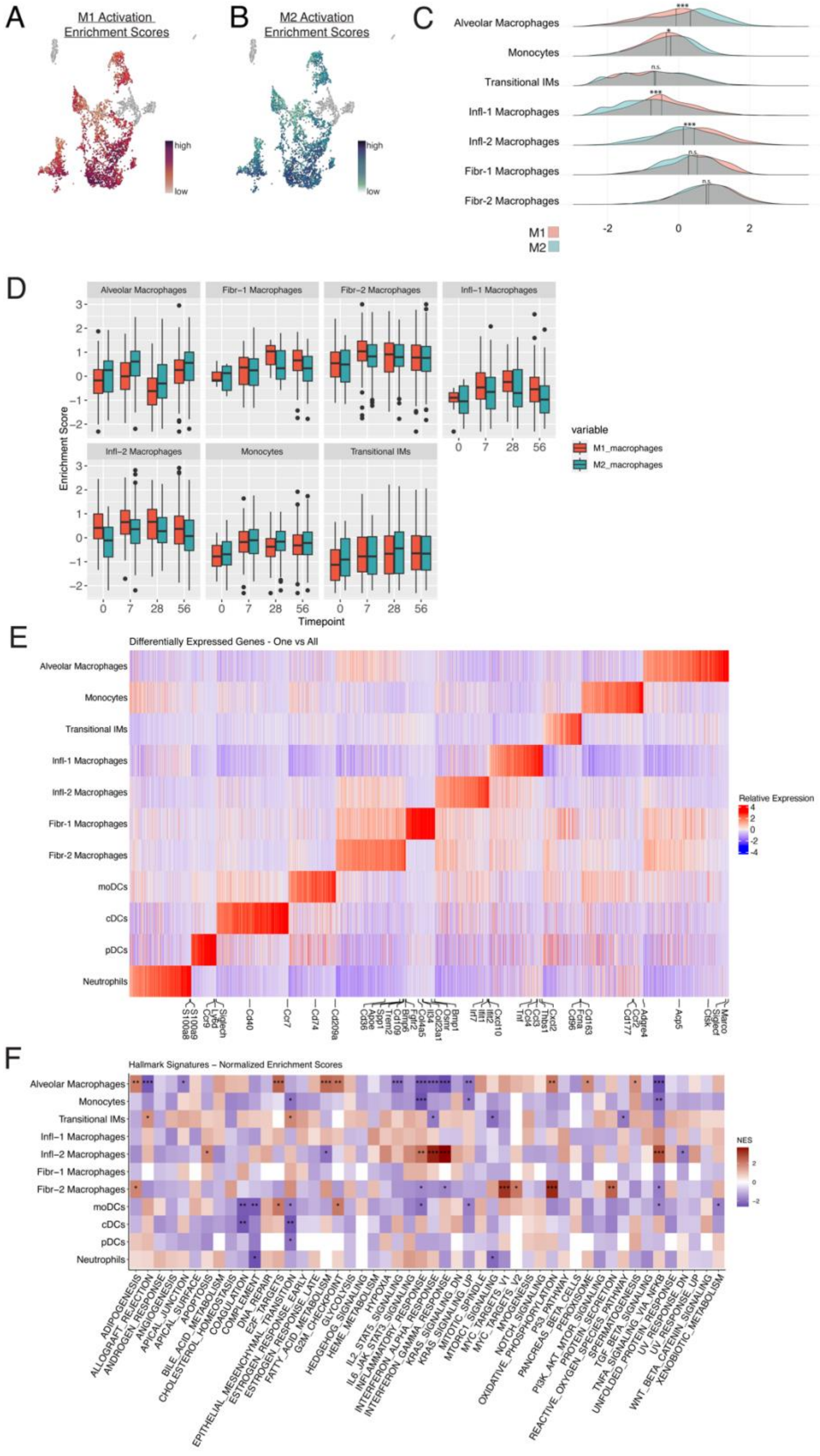

Fig. S3. Analysis of M1 and M2 gene signatures, differentially expressed genes and pathway activation levels to characterize myeloid cell states in silica challenged mice. (**A**) M1 and (**B**) M2 activation scores for each myeloid cell. (**C**) Comparison of M1 and M2 activation enrichment scores for myeloid cells (paired T-test, BH). (D) M1 and M2 gene lists are shown plotted by timepoint for each of the cell states annotated. (E) For each cell state identified from clustering, we identified differentially expressed genes using linear mixed effect models and application of a ‘one cell state vs all others’ approach. Genes were considered significant and retained for the analysis if the adjusted p-value <0.05 in any cell state. Genes were first grouped based on which cell state had the highest coefficient for the DEG and then plotted within these groups in descending order. (F) Normalized enrichment scores for Hallmark pathways demonstrate activation of various biological pathways in each cell state compared to other myeloid cell states. Normalized enrichment scores were calculated with fgsea using all coefficients from the DEG analysis. Significant NES are designated with asterisks (*$p<0.05$, **$p<0.005$, ***$p<0.0005$)

Fig. S4.

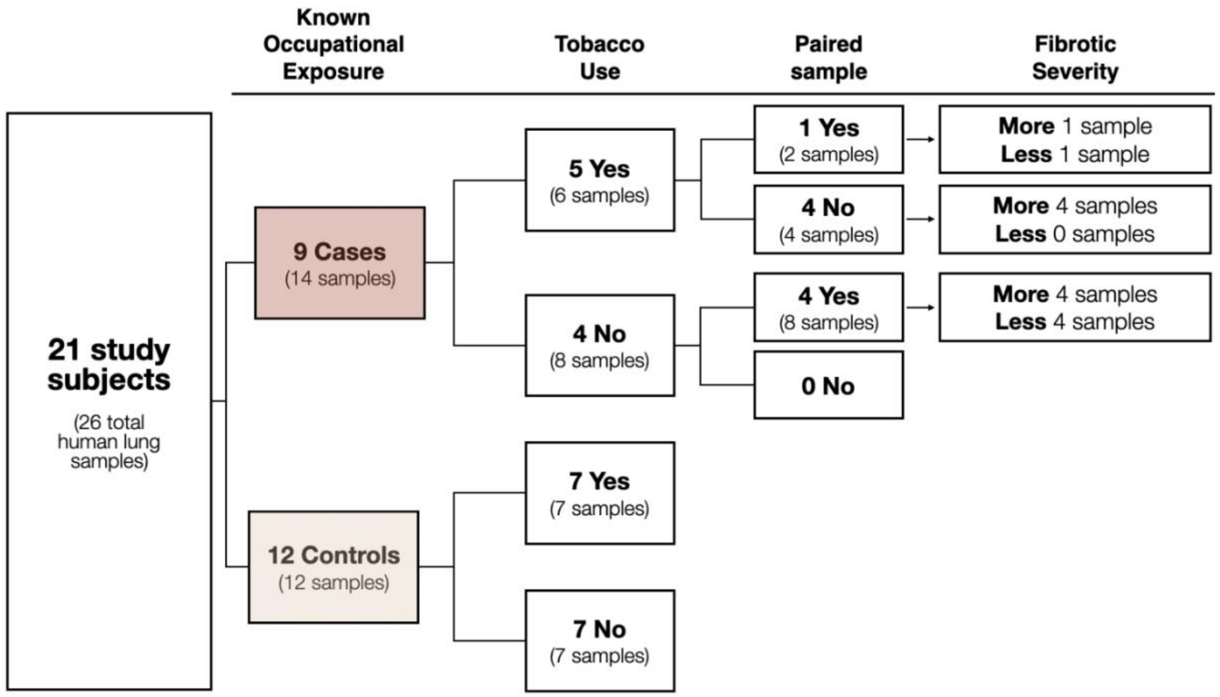

Fig. S4. Schematic of human lung samples used in coal miner single-cell analysis. In total, 26 human lung samples collected from 21 unique individuals were profiled using single-cell RNA-sequencing. Cases and controls were matched based on sex, age, and tobacco use. For some subjects with pulmonary fibrosis, two samples were collected from a more and less fibrotic region of the lung.

Fig. S5.

A. Myeloid cells

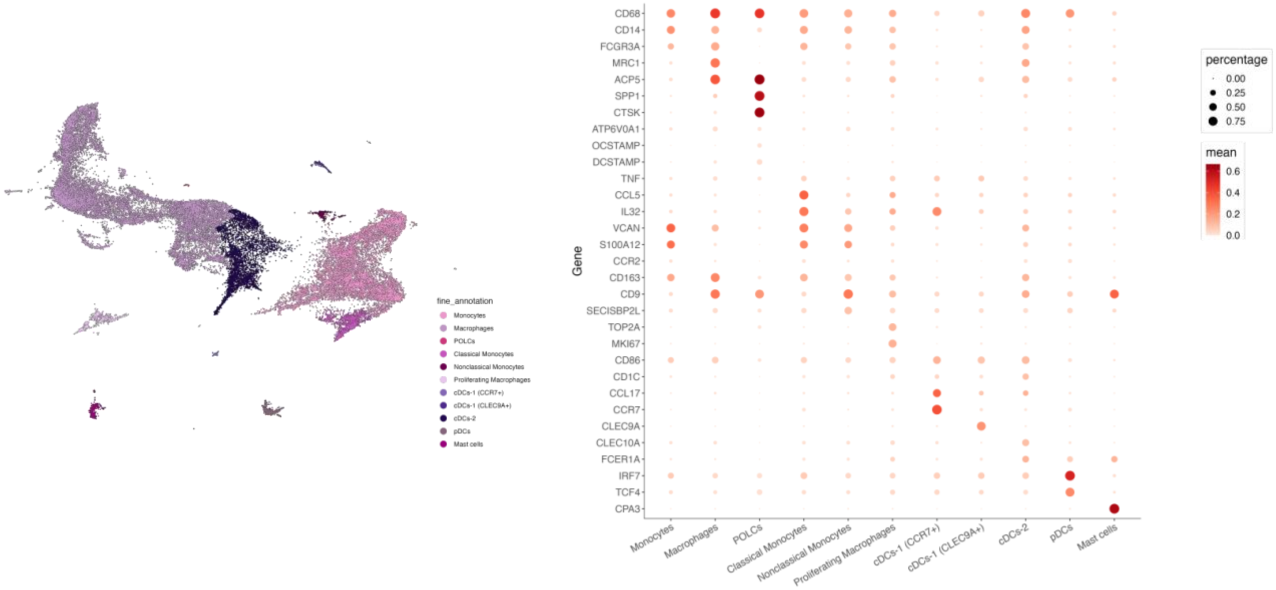

B. Lymphoid cells

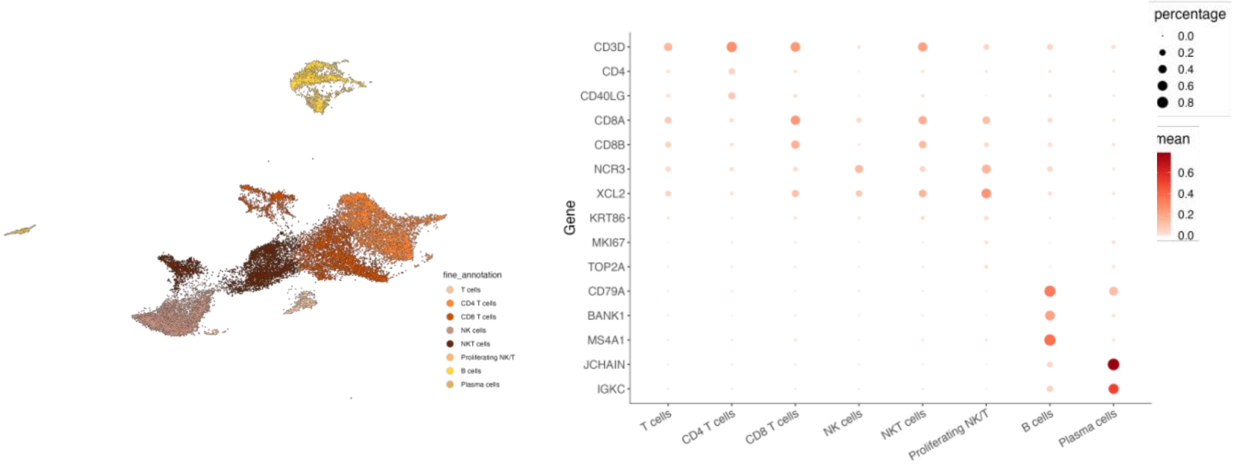

Fig. S5 (cont).

C. Stromal cells

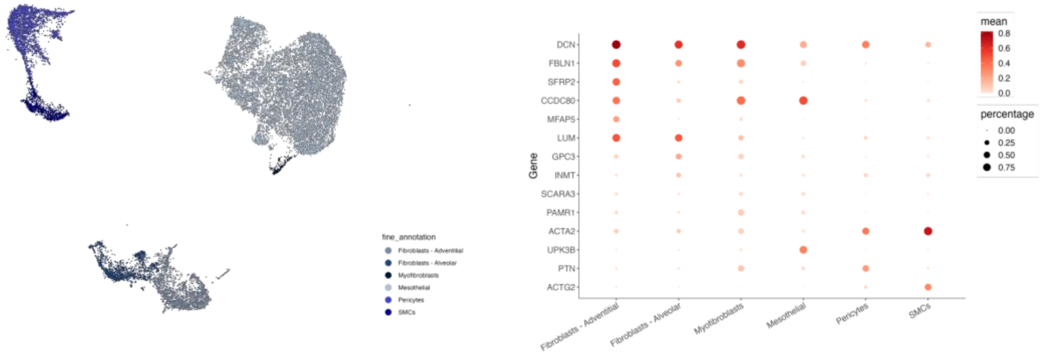

D. Endothelial cells

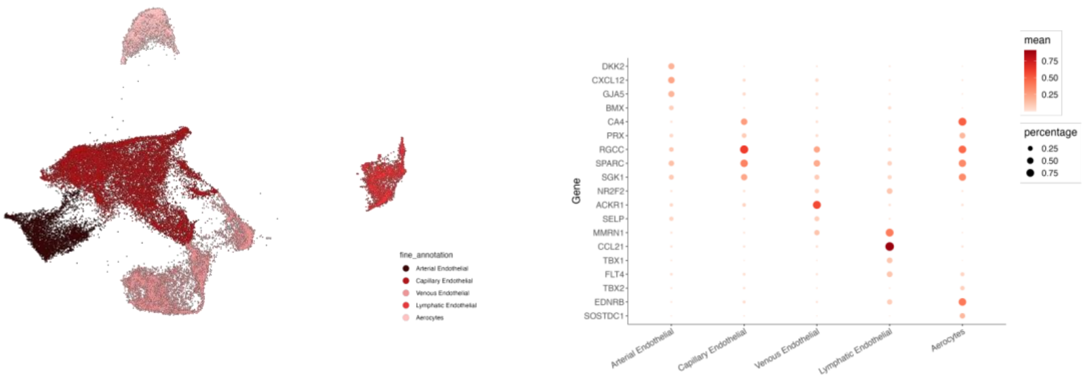

E. Epithelial cells

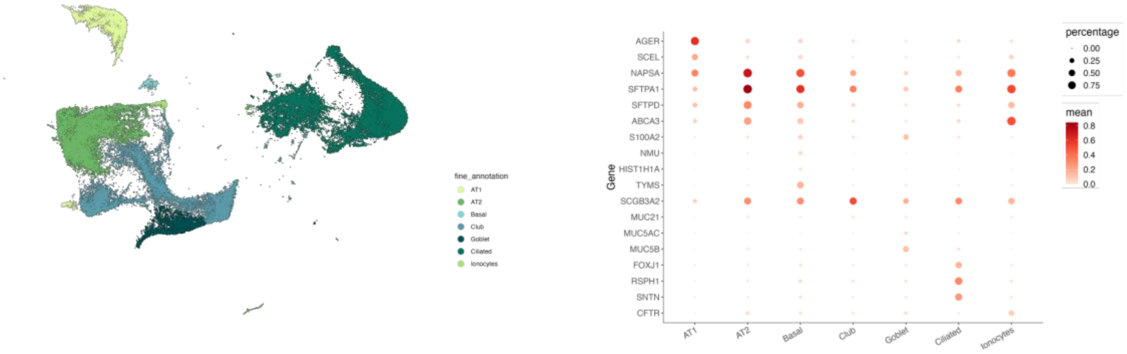

Fig. S5. Fine annotation of single-cell RNA-sequencing data for coal miner lung study. UMAP and gene expression for canonical marker genes are shown for cell types in myeloid (A), lymphoid (B), stromal (C), endothelial (D), and epithelial (E) cell lineages.

Fig. S6

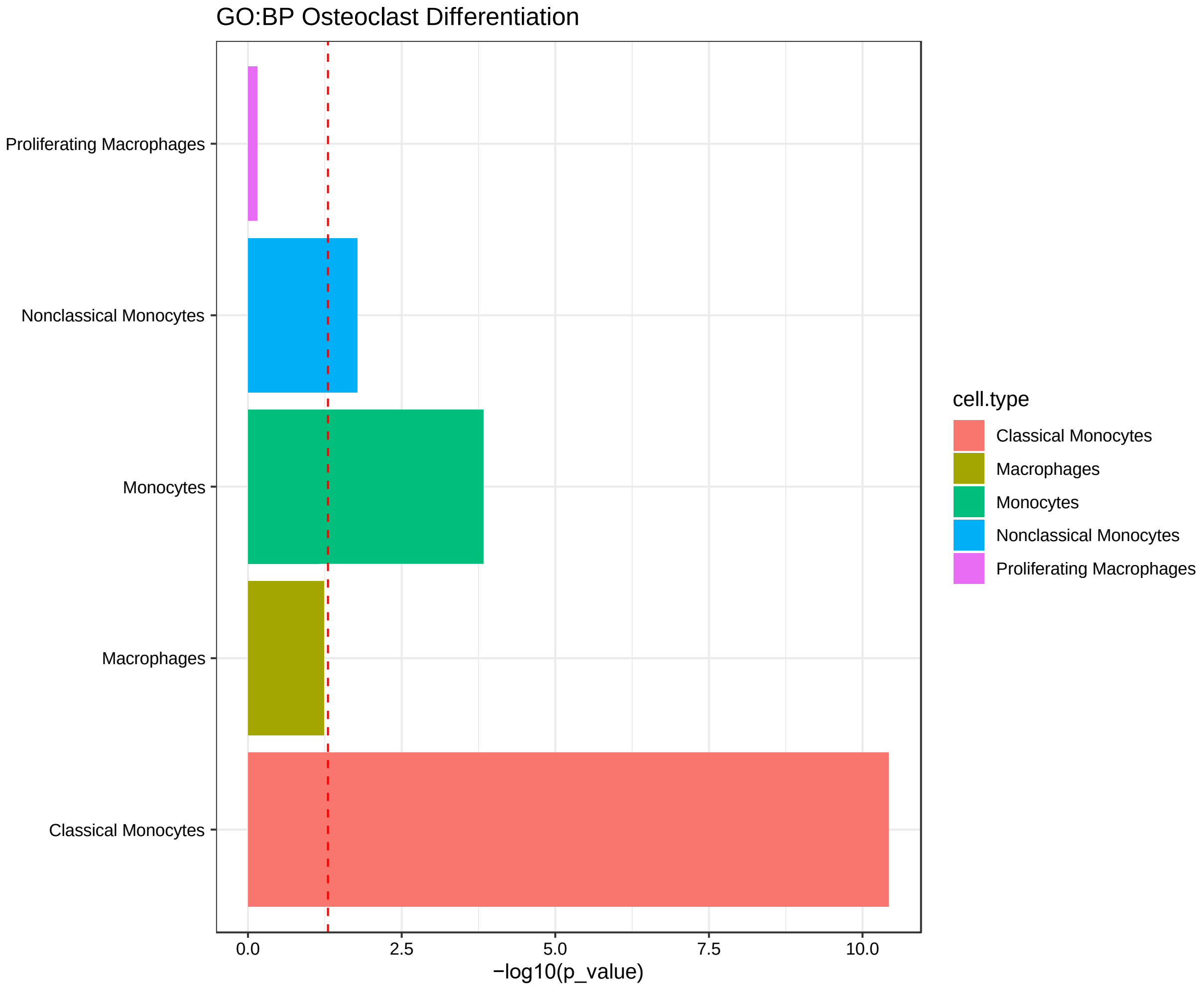

Fig. S6 Osteoclast differentiation is an enriched pathway among DEGs identified between fibrotic silica-exposed human lungs vs non-fibrotic controls for monocyte populations. In this analysis, for each monocyte and macrophage population, we identified DEGs between cases (subjects diagnosed with coal miner pneumoconiosis) vs non-fibrotic controls, controlling for tobacco use. Enriched pathways were identified using gProfiler. Osteoclast differentiation was identified as a significantly enriched pathway among many cell types.

Fig. S7.

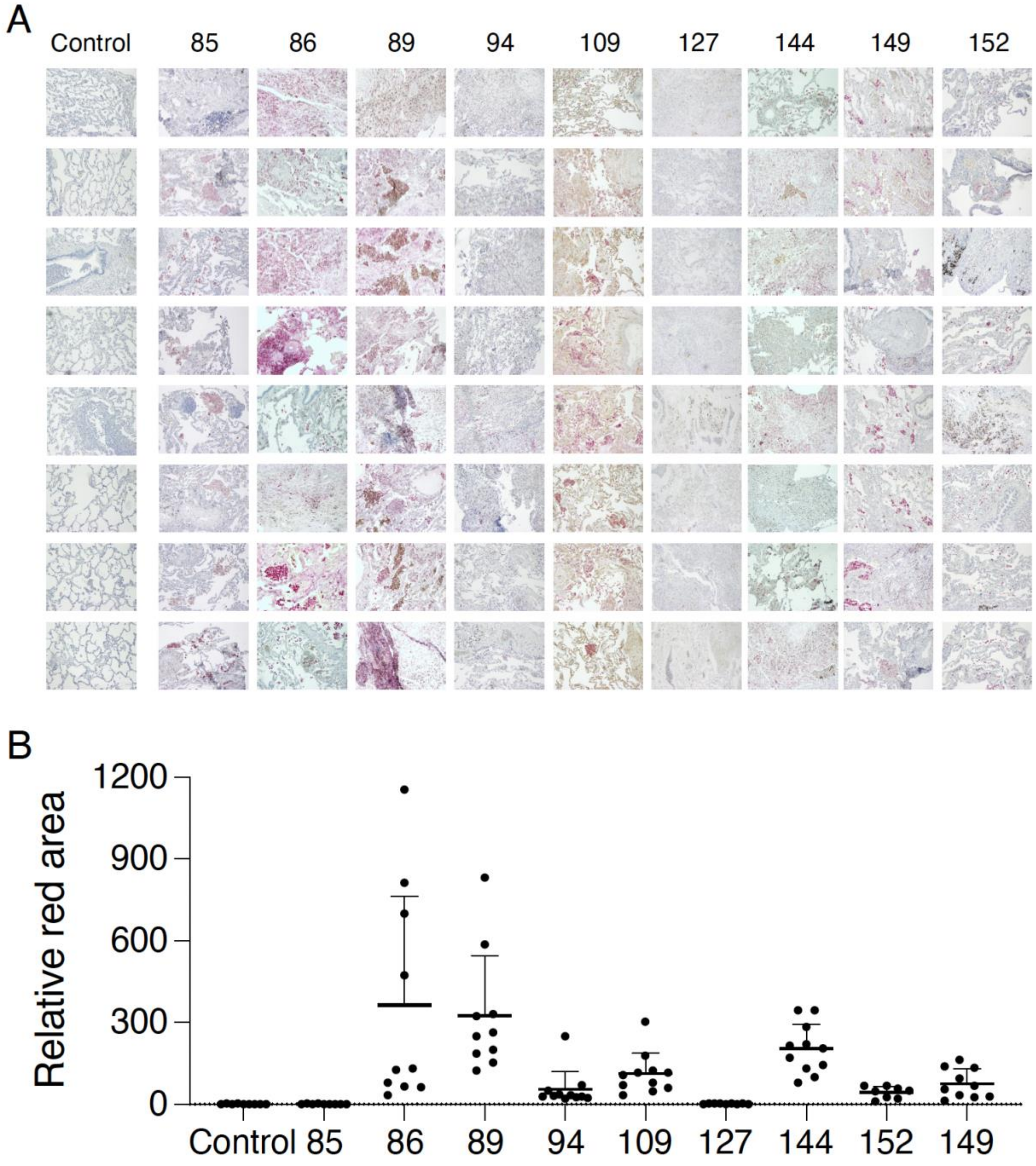

Fig. S7. TRAP and CTSK staining of control and CWP lungs. (A) Lung tissues were collected and stained for CTSK (brown) and TRAP (red) from one control and nine CWP individuals. Images shown are at 100x magnification. (B) 10 random fields for each sample were selected and quantified for TRAP staining using ImageJ.

Fig. S8.

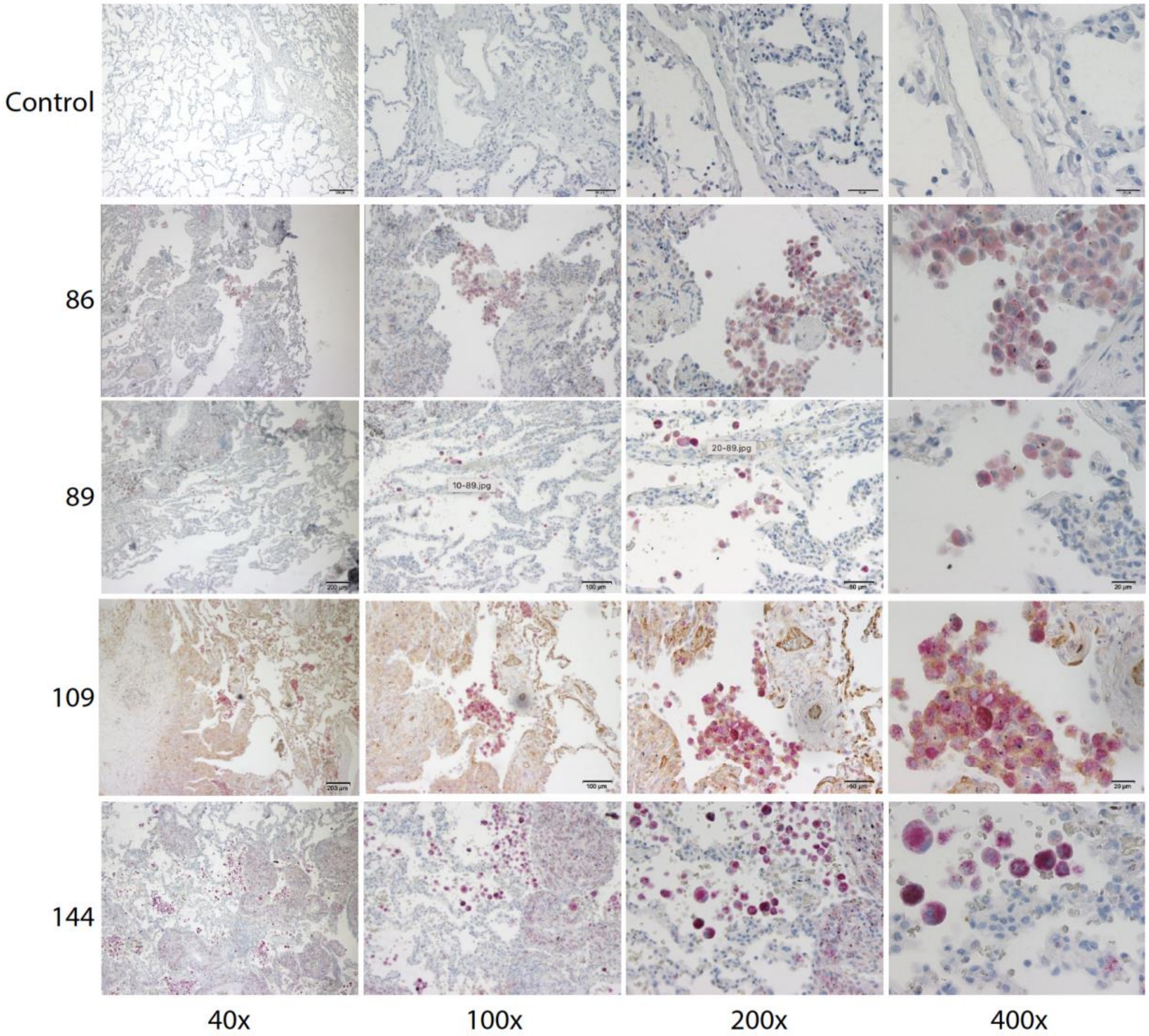

Fig. S8. Higher magnification of TRAP and CTSK staining for control and CWP lung samples.

Fig. S9.

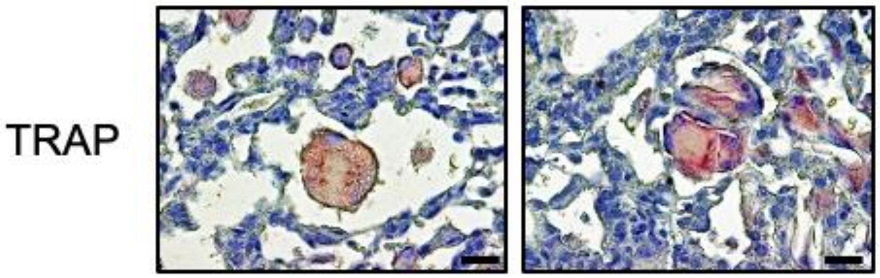

Fig. S9. Silica-induced osteoclast-like cells persist up to 1 year post silica i.t. challenge. Silica particles (5 mg) were administered i.t. into the lungs of C57BL/6J mice. Paraffin-embedded lung sections collected from mice 1 year post silica treatment were stained with TRAP (Scale bar, 20 μm).

Fig. S10.

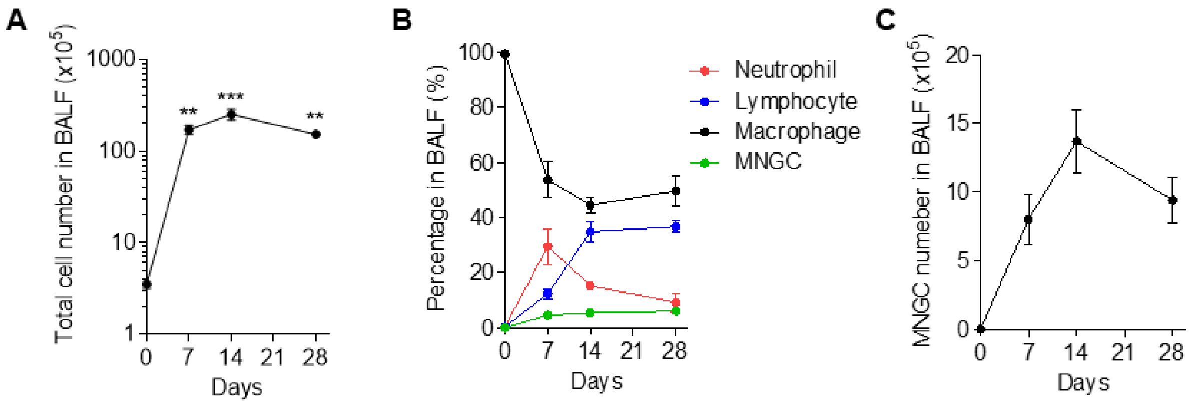

Fig. S10. BAL cell numbers and cell differentiation in i.t. silica challenged mice. Silica particles (5 mg) were administered i.t. into the lungs of C57BL/6J mice. (A) The number of BAL cells at indicated times in days (d) after silica challenge are shown (N = 4 mice per group). (B) The BAL cellular composition at indicated times in days (d) after silica challenge are shown (N = 4 mice per group). (C) The number of MNGC in BALF at indicated times in days (d) after silica challenge are shown (N = 4 mice per group). **P*<0.05, ***P*<0.01, ****P*<0.001

Fig. S11, relates to Fig. 5F

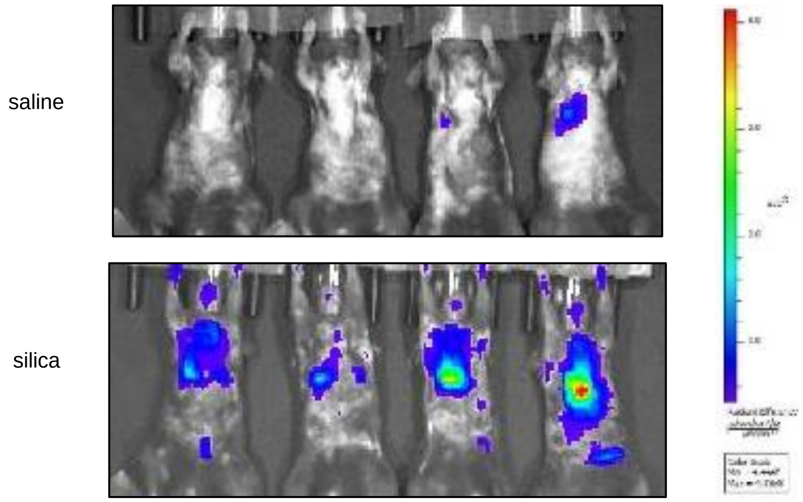

Fig. S11. Intratracheal silica challenge induces CTSK enzyme activity in the lungs of mice. Mice treated with saline or silica i.t. 6 days prior received the cleavage activated fluorescent cathepsin K substrate, Cat K 680 FAST by the i.t. route. Fluorescent images were acquired 18 h later by IVIS and (D) the fluorescent signal was quantified (N = 4 mice per group).

Fig. S12, relates to Fig. 5

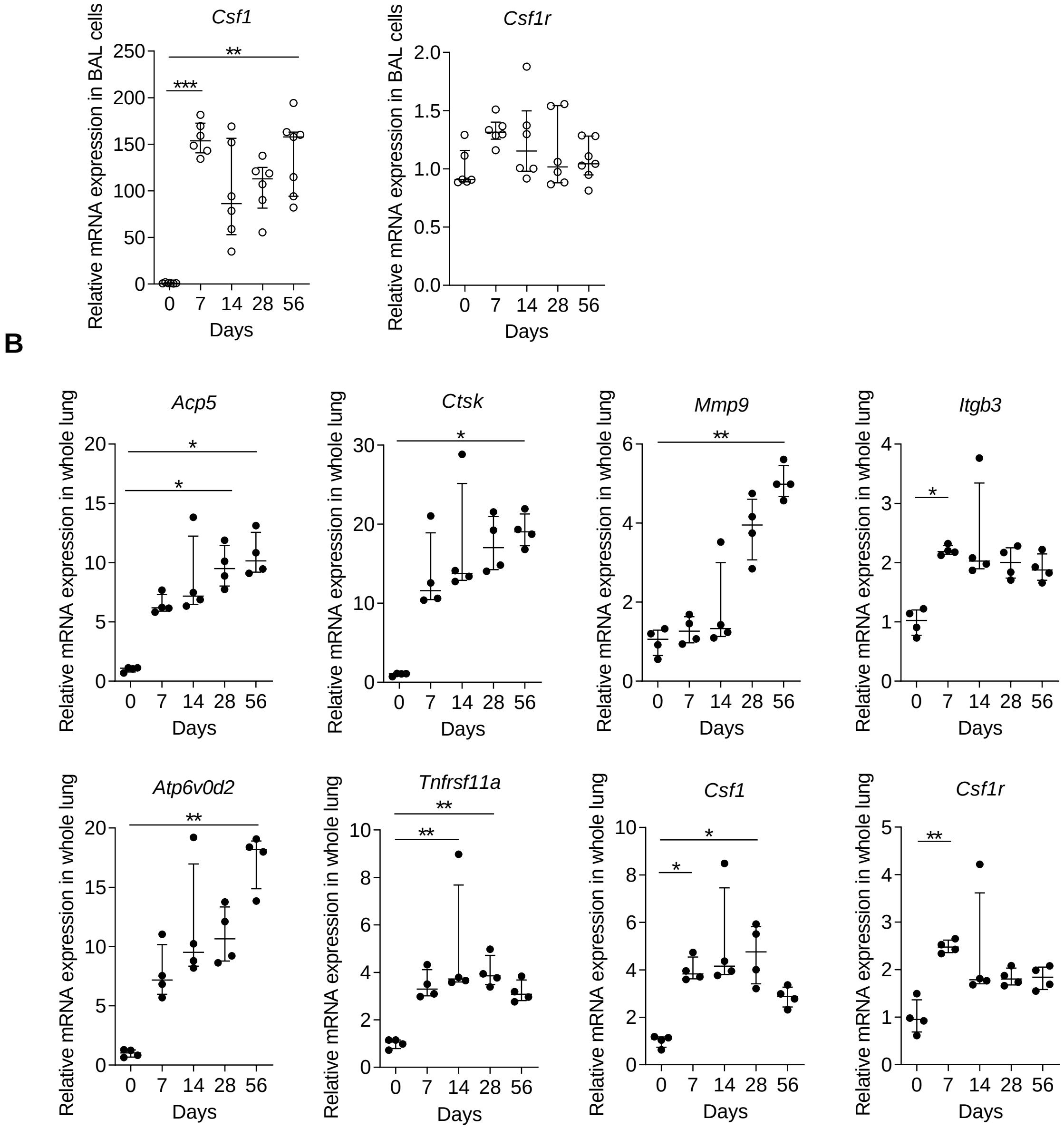

Fig. S12. Osteoclast-related gene expression in BAL cells and lung tissue from silica exposed mice.

Silica particles (5 mg) were administered i.t. into the lungs of C57BL/6J mice. BAL cells (A, white circles) and whole lung tissue (B, black circles) were collected from mice at the indicated times in days (d) after administration, and osteoclast-related gene expression was assessed by rtPCR (N = 6-7 mice per group). Error bars show Median with interquartile range. *$P<0.05$, **$P<0.01$ and ***$P<0.001$ by Mann-Whitney U test in two groups comparison and Kruskal-Wallis test followed by Dunn's test in multiple comparison.

Fig. S13, relates to Fig. 5H and 5I

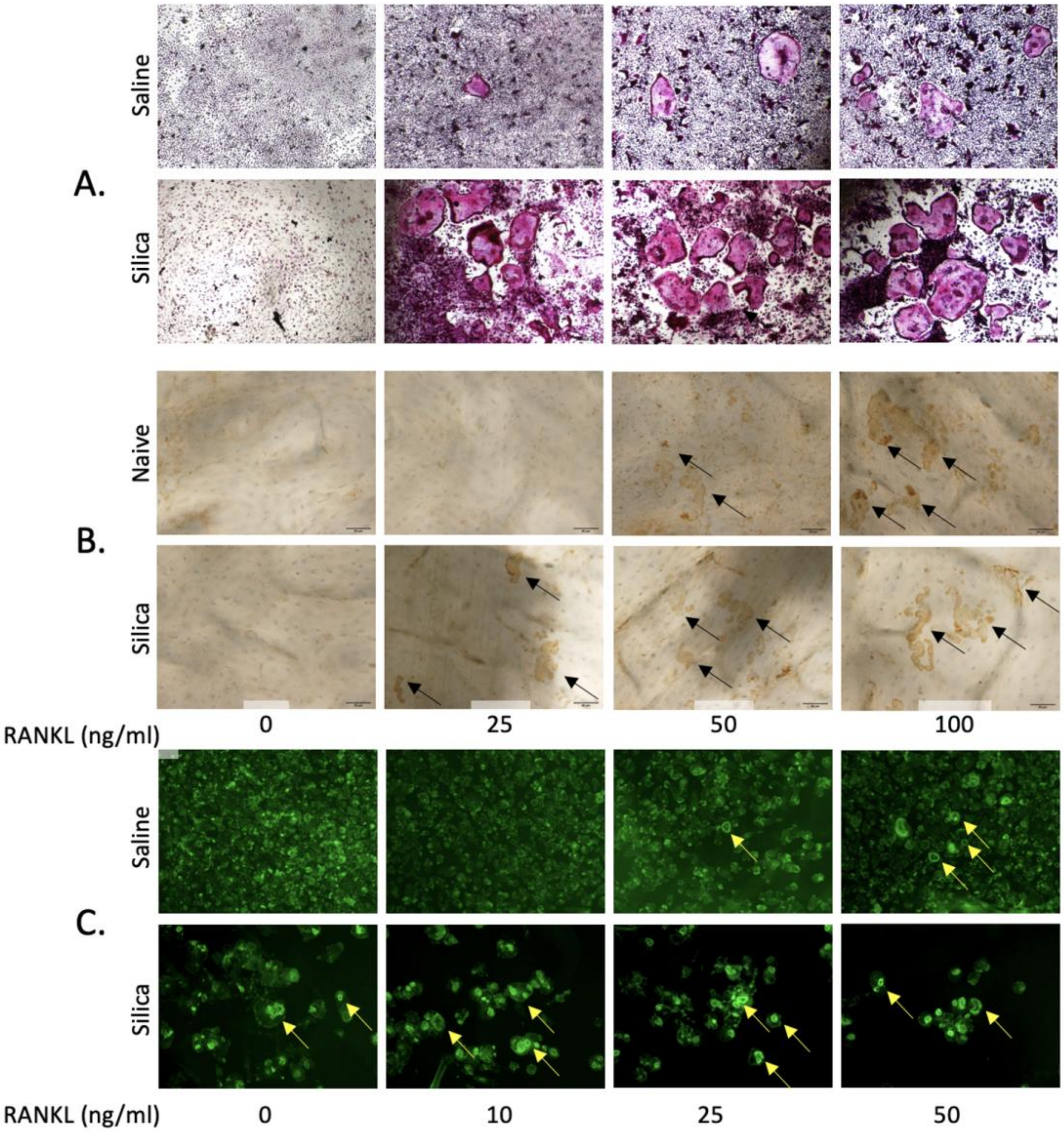

Fig. S13. Intratracheal silica challenge enhances osteoclast formation, bone pitting and actin ring assembly is cultured BAL cells. BAL cells isolated from C57BL/6J mice at day 14 post intratracheal challenge with saline or silica were plated on plastic plates or bovine bone slices with M-CSF and RANKL at indicated concentrations. At day 6, the cells cultured on the plastic plates were stained for TRAP activity (Panel A). For BAL cells plated on bone slices, resorbed bone area was visualized by peroxidase-conjugated wheat germ agglutinin/horse radish peroxidase staining (black arrows, Panel B). Actin rings were visualized by phalloidin staining (yellow arrows, Panel C).

Fig. S14, related to Fig. 5

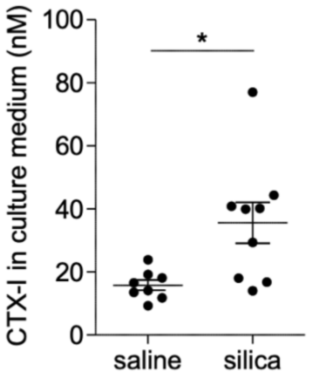

Fig. S14. Intratracheal silica challenge enhances bone matrix degradation by BAL cells cultured on bone slices.
BAL cells isolated from C57BL/6J mice at day 14 post intratracheal challenge with saline or silica were plated on bovine bone slices as above. The levels of CTX-I in bone culture medium were measured by ELISA (The cells were collected from 4 mice in each group and plated cells in 2 wells per mouse). *$P<0.05$, **$P<0.01$, ***$P<0.001$

Fig S15

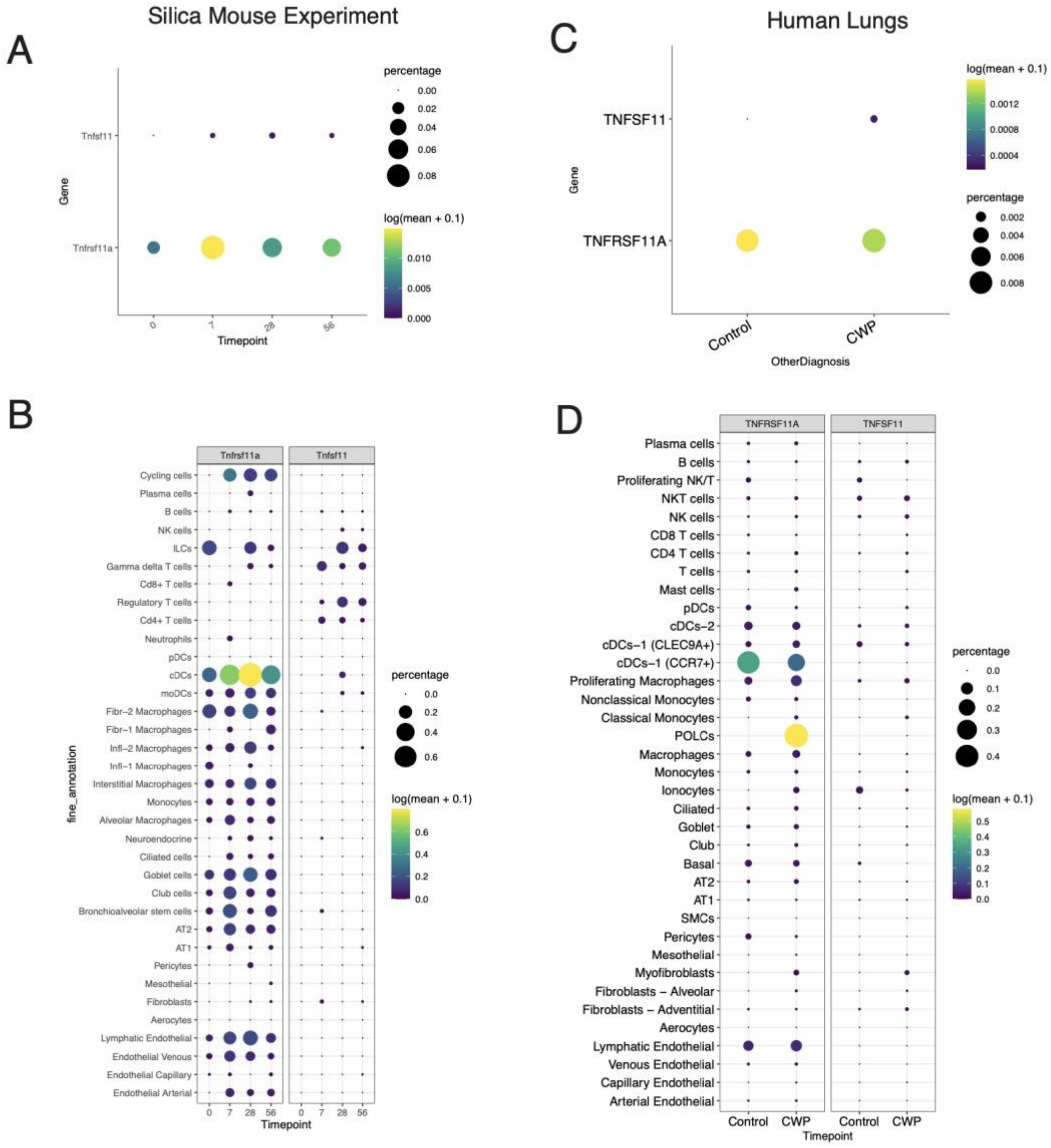

Fig S15 – Expression of RANK and RANKL in silica treated mice and human lung explants. Gene expression values of RANK and RANKL from silicotic mice are plotted over time (A), and by time and cell state (B). Gene expression for RANK and RANKL from human samples are shown stratified by case and control in (C), and case vs control by cell state in (D). Size of the circle represents what percentage of cells have non-zero expression of the gene and color represents the average normalized expression value.

Fig. S16, related to Fig. 6L-O

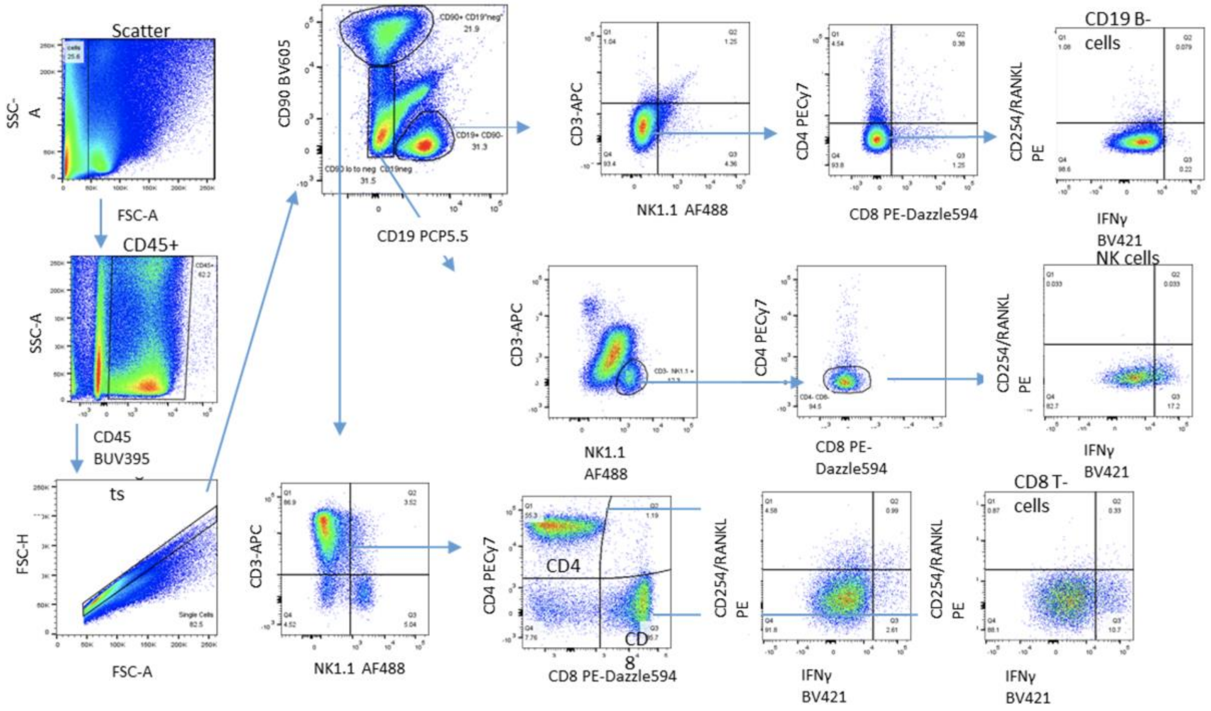

Fig. S16. Flow cytometry gating strategy.
Cells were gated first by a broad gate to exclude debris and aggregates. Leukocytes (CD45+) were then gated followed by a singlet gate (FSC-A vs FSC-H). Single cells were then gated based on expression of the surface markers CD90 and CD19. For populations shown in the manuscript (Extended Data Figure 1, CD19+, CD4+, CD8+, and NK) cells were gated as follows. B-cells were gated first as CD90- CD19+ cells. Subsequent gating was performed to exclude lineage marker expression (CD3 and NK1.1). This was followed by gating for cells negative for CD4 and CD8 expression. These cells were then gated by FSC-A vs SSC-A (not shown) for the PBMC population. The remaining cells were then gated for expression of CD254/RANKL vs INFγ expression. T-lymphocytes were gated initially as CD90+ and CD19-. Lymphocytes were then gated for CD3+ and NK1.1- expression. This was followed by gating for CD4+ vs CD8+ surface expression. These populations were then gated by FSC-A vs SSC-A (not shown) for the PBMC population. The remaining cells were then gated on CD254/RANKL vs INFγ expression. NK cells were gated as CD90- and CD19-, followed by CD3- and NK1.1+ cells. The NK cells without CD4 and CD8 surface expression were then gated on the PBMC population by FSC-A vs SSC-A (not shown). These cells were then assayed for CD254/RANKL vs INFγ expression.

Fig. S17, related to Fig. 6.

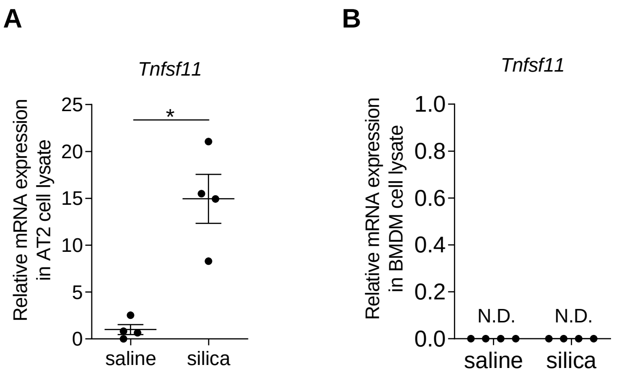

Fig. S17. Silica particles induce RANKL expression in primary rat AT2 cells.
(A) Isolated rat AT2 cells were incubated in DMEM with 10% (v/v) FCS in the presence or absence of 10 µg/ml of silica particles for 3 days. Relative gene expression of Tnfsf11 (RANKL) in AT2 cell lysate was evaluated by rtPCR (N = 4 wells per group). (B) Isolated mouse BMMs were incubated with DMEM in 10% (v/v) FCS and M-CSF (5 ng/ml) in the presence or absence of 10 µg/ml of silica particles for 3 days. Relative gene expression of Tnfsf11 (RANKL) in BMMs was evaluated by rtPCR (N = 4 wells per group) (N.D.; not detected). $^{**}P<0.01$.

Fig. S18, related to Fig. 7B.

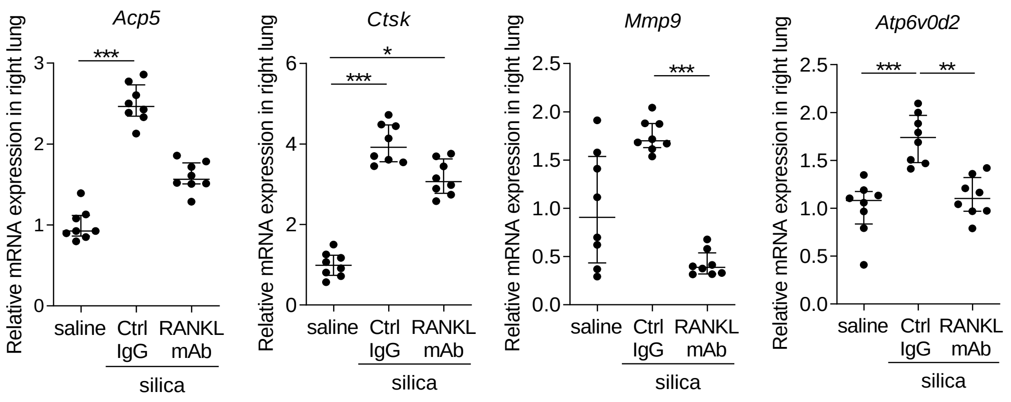

Fig. S18. Osteoclast-related gene expression in lung tissue from silica exposed mice with RANKL mAb treatment.

Mice treated with RANKL mAb or Ctrl IgG (0.25 mg/mouse, i.p., 3 times per week) were challenged with silica (5 mg) and sacrificed 28 days later. RT-qPCR for osteoclast-related genes: Acp5 (TRAP), Ctsk, Mmp9, Atp6v0d2 in whole right lung tissues (N=7-9 mice per group). Error bars show Median with interquartile range. *P<0.05, **P<0.01 and ***P<0.001 by Kruskal-Wallis test following by Dunn's test in multiple comparison.

Fig. S19, related to Fig. 7C.

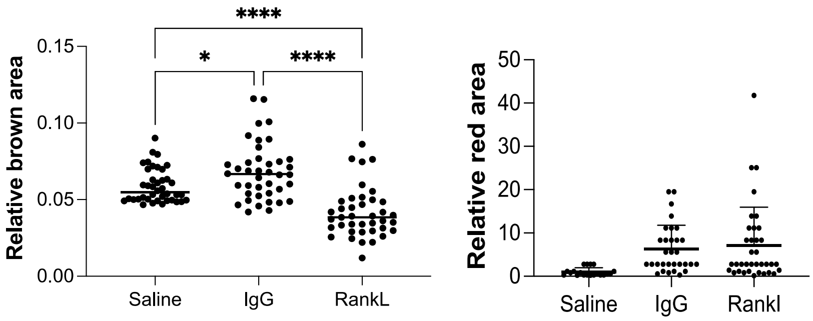

Fig. S19. Semi-quantitative analysis of CTSK (left) and TRAP (right). Staining were analyzed by Nikon NIE CIC Analysis Elements Workstation (NIS-Elements AR, Advanced Research, ver.5). 8-11 random fields were analyzed for TRAP in each sample. 10 random fields were analyzed for CTSK. Fields were quantified for DAB positive staining of CTSK antibody (Abcam) using ImageJ.

Fig. S20, related to Fig. 7F.

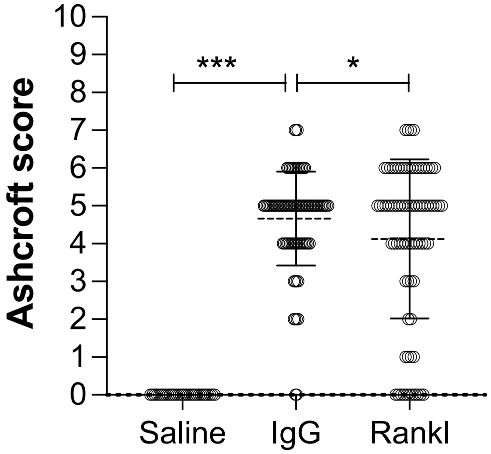

Fig. S20. Ashcroft scoring of tissues indicates significantly reduced burden of fibrosis in silica mice treated with RANKL mAB. Fibrosis lesions were quantified using the Ashcroft score (PMID: 18476815). One H&E stained whole lung section was selected for each mouse and examined at 200x magnification. Fields were randomly selected by serially moving the stage in uniform increments through 63-106 fields, and a blinded second observer scored every third field. In total, 21-35 fields were quantified for each mouse lung were quantified.

Table S1.

	Control (N=12)	CWP (N=9)	Overall (N=21)
**Sex**			
Male	12 (100%)	9 (100%)	21 (100%)
**Age**			
Mean (SD)	55.2 (7.17)	58.7 (7.02)	56.7 (7.15)
Median [Min, Max]	54.5 [41.0, 69.0]	59.0 [47.0, 67.0]	55.0 [41.0, 69.0]
**Race**			
African_American	1 (8.3%)	0 (0%)	1 (4.8%)
European	10 (83.3%)	9 (100%)	19 (90.5%)
Unknown	1 (8.3%)	0 (0%)	1 (4.8%)
**Tobacco**			
No	5 (41.7%)	4 (44.4%)	9 (42.9%)
Yes	7 (58.3%)	5 (55.6%)	12 (57.1%)

Table S1. Study subject characteristics for cases and controls in coal miner single-cell analysis. Cases (coal workers pneumoconiosis) and controls were matched based on age, sex, and tobacco use. In total, 21 subjects were included in the single cell RNA-sequencing experiment.

Table S2.

CWP Pulmonary Function Tests, stratified by Tobacco Use

	No (N=4)	Yes (N=5)	Overall (N=9)
**FEV1**			
Mean (SD)	1.45 (0.798)	1.38 (0.386)	1.41 (0.561)
Median [Min, Max]	1.28 [0.680, 2.57]	1.38 [0.810, 1.77]	1.32 [0.680, 2.57]
**FVC**			
Mean (SD)	2.92 (0.683)	3.00 (0.379)	2.96 (0.498)
Median [Min, Max]	3.17 [1.92, 3.43]	3.18 [2.38, 3.27]	3.18 [1.92, 3.43]
**TLC**			
Mean (SD)	6.08 (0.518)	5.48 (2.04)	5.82 (1.27)
Median [Min, Max]	6.10 [5.58, 6.54]	6.66 [3.13, 6.66]	6.51 [3.13, 6.66]
Missing	0 (0%)	2 (40.0%)	2 (22.2%)
**DLCO.**			
Mean (SD)	50.0 (1.63)	53.3 (26.1)	51.4 (15.2)
Median [Min, Max]	50.0 [48.0, 52.0]	56.0 [26.0, 78.0]	50.0 [26.0, 78.0]
Missing	0 (0%)	2 (40.0%)	2 (22.2%)

Table S2. Pulmonary function test metrics for cases in coal miner single-cell analysis, stratified by tobacco use. All cases represented end-stage lung disease and were collected at time of pulmonary transplant. Reported tobacco was not associated with large differences in pulmonary function.

Table S3.

Gene	Primer	
	Forward	Reverse
Acp5	5’-GCCACAGTTATGTTTGTACGTG-3	5’-ACAGATTGCATACTCTAAGATCTCC-3′
Acta2	5′-CTGTTATAGGTGGTTTCGTGG A-3′	5′-GAGCTACGAACTGCCTGAC-3′
Actb	5′-ACCTTCTACAATGAGCTGCG-3′	5′-CTGGATGGCTACGTACATGG-3′
Atp6v0d2	5’-GCCAAATGAGTTCAGAGTGATG-3’	5’-AGTCTTACCTTGAGGCATTCTAC-3’
Col1α1	5’-CATTGTGTATGCAGCTGACTTC-3’	5’-CGCAAAGAGTCTACATGTCTAGG-3’
Col3α	5‘-TCTCTAGACTCATAGGACTGACC-3’	5’-TTCTTCTCACCCTTCTTCATCC-3’
Csf1	5’-GGAAGATGGTAGGAGAGGGTA-3’	3’-AGGATGAGGACAGACAGGT-5’
Csf1r	5′-AGGTGTAGCTATTGCCTTCG-3′	5′-TGTATGTCTGTCATGTCTCTGC-3′
Ctsk	5’-ATCTCTCTGTACCCTCTGCAT-3′	5’-GACTCTGAAGATGCTTACCCA-3′
Fn1	5’-TTGTTCGTAGACACTGGAGAC-3’	5’-GAGCTATCCATTTCACCTTCAGA-3’
Itgb3	5’-ACAGTCATCCTCGTTCTTGTAG -3′	5’-GAACGCTCCATGAAGAAAACAC-3′
Mmp9	5’-GTGGGAGGTATAGTGGGACA-3′	5’-GACATAGACGGCATCCAGTATC-3′
Tgfb1	5’-CCGAATGTCTGACGTATTGAAGA-3’	5’-GCGGACTACTATGCTAAAGAGG-3’
Tnfsf11	5’-AGTGCTGTCTTCTGATATTCTGT -3′	5′-TCCCGCTCCATGTTCCT-3′
Tnfrsf11a	5’-CACTGTCGGAGGTAGGAGT-3’	5’-CAGGAGAGGCATTATGAGCAT-3’
Tnfrsf11b	5’-ATGCAACACATGACAACGTG-3′	5′-TGGTATAATCTTGGTAGGAACAGC-3′

Table S3. Primers for quantitative RT-qPCR
Acp5, acid phosphatase 5; Acta2, actin α-2 smooth muscle; Actb, β-actin; Atp6v0d2, ATPase H^+ transporting v0 subunit d2; Col1α1, collagen 1α-1; Col3α1, collagen 3α-1; Csf1, colony stimulating factor 1; Csf1r, Csf1 receptor; Ctsk, cathepsin K; Fn1, fibronectin 1; Itgb3, Integrin β3; Mmp9, matrix metallopeptidase 9; Tgfb1, transforming growth factor-β1; Tnfsf11, tumor necrosis factor receptor superfamily member 11; Tnfrsf11a , tumor necrosis factor receptor superfamily member 11a; Tnfrsf11b, tumor necrosis factor receptor superfamily member 11B.

Table S4.

s.genes	g2m.genes
MCM5	HMGB2
PCNA	CDK1
TYMS	NUSAP1
FEN1	UBE2C
MCM2	BIRC5
MCM4	TPX2
RRM1	TOP2A
UNG	NDC80
GINS2	CKS2
MCM6	NUF2
CDCA7	CKS1B
DTL	MKI67
PRIM1	TMPO
UHRF1	CENPF
MLF1IP	TACC3
HELLS	FAM64A
RFC2	SMC4
RPA2	CCNB2
NASP	CKAP2L
RAD51AP1	CKAP2
GMNN	AURKB
WDR76	BUB1
SLBP	KIF11
CCNE2	ANP32E
UBR7	TUBB4B
POLD3	GTSE1
MSH2	KIF20B
ATAD2	HJURP
RAD51	CDCA3
RRM2	HN1
CDC45	CDC20
CDC6	TTK
EXO1	CDC25C
TIPIN	KIF2C
DSCC1	RANGAP1
BLM	NCAPD2

CASP8AP2	DLGAP5
USP1	CDCA2
CLSPN	CDCA8
POLA1	ECT2
CHAF1B	KIF23
BRIP1	HMMR
E2F8	AURKA
	PSRC1
	ANLN
	LBR
	CKAP5
	CENPE
	CTCF
	NEK2
	G2E3
	GAS2L3
	CBX5
	CENPA

Table S4. Gene lists used for calculating proliferation index. Proliferation index is calculated as the log sum of normalized aggregated expression value for each list. For sn-RNAseq data from mouse, the R package biomaRt was used to translate human genes into mouse orthologs.
